# Replacement of Saturated Fatty Acids from Meat by Dairy Sources in Relation to Incident Cardiovascular Disease: The European Prospective Investigation into Cancer and Nutrition (EPIC)-Norfolk Study

**DOI:** 10.1016/j.ajcnut.2024.04.007

**Published:** 2024-04-11

**Authors:** Yakima D Vogtschmidt, Sabita S Soedamah-Muthu, Fumiaki Imamura, David I Givens, Julie A Lovegrove

**Affiliations:** 1 Hugh Sinclair Unit of Human Nutrition, Department of Food and Nutritional Sciences, University of Reading, Reading, United Kingdom; 2 Institute for Cardiovascular and Metabolic Research, University of Reading, Reading, United Kingdom; 3 Institute for Food, Nutrition and Health, University of Reading, Reading, United Kingdom; 4 Center of Research on Psychological Disorders and Somatic Diseases (CoRPS), Department of Medical and Clinical Psychology, Tilburg University, Tilburg, Netherlands; 5 Medical Research Council Epidemiology Unit, University of Cambridge School of Clinical Medicine, Cambridge, United Kingdom

**Keywords:** adults, cardiovascular disease, cheese, dairy, meat, milk, prospective study, replacement, saturated fatty acids, yogurt

## Abstract

**Background:**

Prospective observational data revealed lower cardiovascular disease (CVD) incidence with modeled replacement of saturated fatty acids (SFA) from total meat by total dairy, but it is unknown what the associations are of replacing SFA from types of meat by types of dairy with CVD incidence.

**Objectives:**

This study aimed to investigate the associations of replacing SFA from total, red, processed, and poultry meat by SFA from total dairy, milk, cheese, and yogurt with the incidence of CVD.

**Methods:**

We analyzed longitudinal data from 21,841 participants of the European Prospective Investigation into Cancer and Nutrition-Norfolk study (56.4% female; age, 40–79 years). Dietary data were collected by food frequency questionnaires at baseline (1993–1997). Incident fatal or nonfatal CVD (*n* = 5902), coronary artery disease (CAD; *n* = 4215), stroke (total: *n* = 2544; ischemic: *n* = 1113; hemorrhagic: *n* = 449) were identified up to 2018. Hazard ratios (HR) and 95% confidence intervals (CI) were estimated using Cox regression for the risk associated with replacement of 2.5% of energy from SFA from meat by dairy, adjusted for sociodemographic, lifestyle, energy, dietary, and cardiometabolic factors.

**Results:**

Replacing SFA from total meat by total dairy was associated with a lower CVD incidence (HR: 0.89; 95% CI: 0.82, 0.96) and CAD (HR: 0.88; 95% CI: 0.80, 0.96). Replacing SFA from processed meat by cheese was associated with lower CVD (HR: 0.77; 95% CI: 0.68, 0.88); CAD (HR: 0.77; 95% CI: 0.66, 0.90), and stroke (HR: 0.81; 95% CI: 0.67, 0.99). Similarly, replacing SFA from red meat by cheese was associated with lower CVD (HR: 0.86; 95% CI: 0.76, 0.97). Higher incidence of stroke was found with replacement of SFA from poultry by milk (HR: 2.06; 95% CI: 1.09, 3.89), yogurt (HR: 2.55; 95% CI: 1.27, 5.13), or cheese (HR: 1.96; 95% CI: 1.04, 3.70), but the CI were relatively large, owing to low, narrow range of poultry SFA intake.

**Conclusions:**

Findings indicate that different SFA-rich foods at baseline have differential associations with CVD risk. If confirmed by further studies, these findings could be used to inform specific food-based dietary guidance.

## Introduction

Cardiovascular diseases (CVD), including coronary artery disease (CAD) and stroke, are a leading cause of mortality and major cause of disability worldwide [1]. Prevalent cases of CVD were estimated to be 523 million people, and the total number of deaths has reached 18.6 million globally in 2019 [1]. Additionally, CVD is an important cause of premature death, contributing to 6.2 million mortality cases in the age category of 30–70 y in 2019 [1], adding to the need to identify potential modifiable risk factors, including diet, to prevent and manage CVD.

Current dietary recommendations to reduce CVD risk include limiting SFA intake to 10% of total energy intake (en%) and suggest limiting the intake of meats, whereas increasing the intake of other animal-based and plant-based foods [2,3]. Some prospective cohort studies have used replacement models to investigate the associations of replacing SFA from total meat by SFA from total dairy with the incidence of total CVD and/or CAD [4,5]. The Multiethnic Study of Atherosclerosis study showed 25% and 24% lower incidence of CVD and CAD, respectively, with the replacement of 2 en% of SFA from total meat by the same amount of energy of SFA from total dairy [4]. This is consistent with uresults from the European Prospective Investigation into Cancer and Nutrition (EPIC)-Netherlands study, showing a 6% lower incidence of CAD with replacement of 1 en% of SFA from total meat by SFA from total dairy [5].

Although these cohort studies suggest inverse associations with the incidence of CVD and CAD following the replacement of SFA from meat by dairy products, the findings are based on 2 studies only [4,5], conducted among United States and Dutch adults, with no equivalent evidence for the UK adult population. Additionally, total meat and total dairy were evaluated, not individual meat or dairy types, which might have differential associations with CVD risk. To illustrate, a recent review suggests detrimental associations for processed meat intake with CVD (including CAD and stroke); varying associations for (unprocessed) red meat intake; and nonsignificant or inverse associations for white meat intake [6]. For individual dairy products, cheese consumption was found associated with CVD, CAD, and stroke, without clear evidence of the dose–response relationship [7]. No associations were observed for milk and yogurt intake [7]. Furthermore, uncertainties remain for stroke and its subtypes, which were not analyzed as separate outcomes. Large United States prospective cohorts reported lower risk of stroke with replacement of 1 serving/d of red meat with 1 serving/d of low-fat or high-fat dairy [8]. However, these replacements were performed on food-intake level, not iso-energetically, which might be prone to confounding resulting from (unspecified) residual energy replacement (owing to differences in energy content of the foods).

This study examined the isoenergetic replacement of SFA from total and individual types of meat by SFA from total and individual types of dairy in relation to the incidence of CVD, CAD, and stroke in the UK population. We also examine subtypes of stroke as previous evidence suggests differences in the associations between dairy foods and stroke subtypes [9], which may be due to differences in etiology, some risk factors, and the number of cases in the cohort [9].

## Methods

### Study design and population

A complete-case analysis was performed, using data from the EPIC-Norfolk study. The rationale and design of this study has been reported previously [10,11]. The EPIC-Norfolk study is a UK population-based cohort study of 25,639 males and females, aged 40–79 years, recruited through general practices in the Norfolk area between 1993 and 1997 (baseline). All participants provided written informed consent, and the study was approved by the Norfolk Research Ethics Committee. Participants without a prevalent CVD, with plausible energy intakes and with complete data at baseline were prospectively analyzed in this study (*n* = 21,841) (Supplemental Figure 1).

### Dietary assessment

Data on daily intake of SFA from different food items and other nutrients were estimated from a semiquantitative 130-item food frequency questionnaire (FFQ) at baseline [12] using FFQ EPIC Tool for Analysis Software [13]. Participants were asked to report how often, on average, they have consumed each food item during the past year according to response categories, ranging from “never or less than once a month” to “6+ per day” [12]. Data on the type of milk were collected via the question: “What type of milk did you most often use?” with response options of “full cream/whole,” “skimmed,” “dried milk,” “semi-skimmed,” “channel islands, gold,” “soya,” “other, specify,” and “none.” Additional questions were asked on the total quantity of milk consumed per day with options ranging from “none” to “more than 1 pint (568 mL).” Spearman correlation coefficients between the FFQ and 16-day weighted records were 0.55 and 0.56 for the estimated intakes of total fat and saturated fat, respectively [14,15]. Definitions of the meat and dairy food groups can be found in Supplemental Table 1.

### Outcome ascertainment

Incident cases of fatal or nonfatal CVD during follow-up were identified using the national hospital record linkage system (East Norfolk Commission Record) and death certificates from the UK’s Office of National Statistics. CVD events included CAD or stroke [International Statistical Classification of Diseases and Related Health Problems, 10th revision (ICD-10): I20–I25, I60–I69]. CAD events were defined through ICD-10 codes I20–I25. Stroke events were defined as the incidence of ischemic, hemorrhagic, and stroke of unspecified cause (ICD-10 codes: I60–I69). A previous validation study in a subset of incident cases of stroke within the EPIC-Norfolk suggested that hospital record linkage and death certificates were valid methods to identify true incident cases of stroke, with sensitivity of 94% [16].

### Covariates

Covariates included sex, age, educational level, physical activity status, smoking status, use of hormone replacement therapy (females only), and presence of self-reported diabetes and were collected using Health Lifestyle Questionnaires [17] at baseline. Data on BMI (in kg/m^2^), waist circumference (in centimeters), and blood pressure (in millimetres of mercury) were obtained from clinical measurements at baseline. Waist circumference was measured using a D-loop nonstretch fiberglass tape at the smallest circumference between the ribs and iliac crest while the participant was standing straight. Systolic and diastolic blood pressure values were measured twice using an Accutorr sphygmomanometer, after seated resting for 5 min. The mean of the 2 measurements were used for analysis. Baseline hypertension was defined as systolic blood pressure of ≥140 mm Hg or diastolic blood pressure of ≥90 mm Hg or self-reported use of antihypertensive medication or high-blood pressure at baseline. Baseline hypercholesterolemia was defined as self-reported high cholesterol or use lipid-lowering medication at baseline. Participants were asked if they could report the presence of high blood cholesterol at baseline. The response categories were “yes” or “no.”

### Statistical analyses

The intakes of SFA were expressed as percentage of total energy intake (en%). Baseline intakes of SFA from total dairy, milk, cheese, total meat, red meat, processed meat, and poultry were categorized into quintiles, whereas SFA intakes from yogurt were categorized into tertiles. Correlation analyses (between the estimates in energy and mass of SFA) were performed to evaluate the extent to which we can separate the impact of food-specific SFA from the food group itself and the food-specific SFA estimates from each other.

For longitudinal analysis, person-years was defined as the time between the date of study entry at baseline and the date of first event of the outcome of interest or death or end of follow-up (31 March 2018), whichever occurred first. Cox proportional hazard regression models were used to compute the hazard ratios (HR) and 95% CI that represented estimates of the associations of SFA from meat and dairy. The HRs (95% CI) were further estimated calculating the difference between the β coefficients and the respective variance and covariance matrices, for the hypothetical effect of replacing 2.5 en% from meat SFA with 2.5 en% from dairy SFA on the incidence of CVD outcomes. More detailed information on modeled food replacements can be found elsewhere [18,19]. A replacement amount of 2.5 en% was chosen, consistent with UK dietary surveys. The baseline median intake of total SFA was 12.8 (SD: 3.3) en%, which is ∼2.5 en% above the UK recommendation for SFA intake. Visual inspection of the log–log plots did not show clear violation of the proportionality assumption. Martingale residuals plotted against SFA from total meat and total dairy showed no violation of the linearity assumption.

Multivariable-adjusted Cox model included age (in years; continuous); sex; educational level (no, ordinary level, advanced level, and degree; categorical); physical activity (inactive, moderately inactive, moderately active, and active; categorical); smoking status (current, former, and never; categorical); alcohol intake (in grams per day; 2-restricted cubic spline terms, 3 knots); fiber intake (in grams per day; continuous); fruit and vegetables (in grams per day; quintiles); hormone replacement therapy (yes/no; dichotomous, females only); BMI (in kg/m^2^; continuous); waist circumference (in centimeters; continuous); baseline hypertension (yes/no; dichotomous); baseline hypercholesterolemia (yes/no; dichotomous); baseline diabetes mellitus (yes/no; dichotomous); and dietary intakes of cholesterol (in milligrams per day; continuous), *trans-*fatty acids, protein, carbohydrate, PUFA, MUFA, and SFA from the other foods (all expressed as en%; continuous). SFA from the other foods was calculated as the sum of SFA intake from fats and oils (including butter, margarine, added fats, and vegetable oils); cereal and cereal products; sugar; preserves and snacks; potatoes; eggs and egg dishes; fish and fish products; soups and sauces; and vegetables. We used a nutrient density model [19], in which SFA intake was examined as proportion of total energy intake. Other macronutrients, such as *trans*-fatty acids, protein, carbohydrate, PUFA, MUFA, and SFA from other foods were also included and expressed as nutrient density terms. Total energy intake was not included in the above-described multivariable model because dietary cholesterol, *trans*-fatty acids, protein, carbohydrates, PUFA, MUFA, and SFA (from other foods) are sources of total energy intake and, therefore, introduce collinearity. The HR and 95% CI derived from the unadjusted and other adjusted Cox models were also computed. Potential confounders were primarily selected based on existing evidence [4,5], biological plausibility, and statistical efficacy. Association analyses without replacements were also conducted, and results for SFA from each meat or dairy source were mutually adjusted for each other.

Sensitivity analyses included repeated analysis using age as underlying time scale. To examine potential reverse causality, analyses were repeated by excluding participants with self-reported diabetes and cancer at baseline and by excluding the first 2 y of follow-up. Test of interaction was performed by including product terms of the exposure with the following variables in the models: sex (males/females), age (<60/≥60 y), and baseline comorbidity (yes, no/unknown). Stratified analyses were also performed to assess whether the associations were consistent across subgroups. Analyses were conducted using Stata, version 16.1, and version 17.0. *P* values (2-sided) of <0.05 were considered as statistically significant.

## Results

In our study population of 21,841 participants, the mean ± SD age was 58.5 ± 9.2 years, 56.4% were female, the mean ± SD BMI was 26.2 ± 3.9 kg/m^2^, and 99.4% were White. Baseline mean ± SD intake of SFA from total dairy was 3.3 ± 1.9 en%; SFA from total meat was 1.8 ± 1.1 en%. The main dairy sources of SFA were milk (mean ± SD: 1.7 ± 1.6 en%), yogurt (mean ± SD: 0.1 ± 0.3 en%), and cheese (mean ± SD: 1.1 ± 0.9 en%). The main sources of SFA within the meat group were red meat (mean ± SD: 0.8 ± 0.7 en%), processed meat (mean ± SD: 0.7 ± 0.7 en%), and poultry (mean ± SD: 0.2 ± 0.2 en%).

Correlations were weak between the estimates of SFA from total and individual types of meat and total and individual types of dairy, ranging from −0.21 to 0.09 (Supplemental Table 2). Correlations of estimates of SFA from total and individual meat products or from total and individual dairy products with total SFA were weak to moderate (range: −0.24 to 0.53) (Supplemental Table 2). Correlations of SFA estimates from each meat or dairy source with estimates from the corresponding food groups were moderate to high (range: 0.45–1.00) (Supplemental Table 2).

In unadjusted comparisons across quintiles of SFA from total dairy, higher intake was associated with an older age, higher likelihood of having a higher educational degree, and higher likelihood of being current smokers (Table 1). Higher dairy SFA intake was generally associated with more favorable cardiometabolic risk profile and lifestyle behaviors, including lower BMI, lower likelihood of having hypercholesterolemia, and lower intake of alcohol. Among female participants, those with higher intake of dairy SFA were less likely to take hormone replacement therapy. With respect to dietary variables, higher dairy SFA intake was associated with lower intake of meat, fruit and vegetables, fiber, carbohydrate, and PUFA and higher intake of total fat, dietary cholesterol MUFA, *trans*-fatty acids, and total SFA.

**TABLE 1:** Baseline characteristics across quintiles of SFA intake from total dairy (expressed as percentage of total energy intake): 1993–1997, EPIC-Norfolk Study (*N* = 21,841)^1^.

	Q1 (*n* = 4369)	Q2 (*n* = 4368)	Q3 (*n* = 4368)	Q4 (*n* = 4368)	Q5 (*n* = 4368)
Range of SFA from total dairy (en%)	0.0–1.7	1.7–2.6	2.6–3.4	3.4–4.6	4.6–18.1
Age (y)	57.7 ± 8.9	58.1 ± 9.2	58.5 ± 9.2	59.0 ± 9.3	59.4 ± 9.4
Sex^2^, female	2558 (58.6)	2431 (55.7)	2455 (56.2)	2475 (56.7)	2398 (54.9)
Educational level^2^					
Ordinary level	445 (10.2)	489 (11.2)	443 (10.1)	471 (10.8)	451 (10.3)
Advanced level	1745 (39.9)	1720 (39.4)	1862 (42.6)	1756 (40.2)	1717 (39.3)
Degree	508 (11.6)	536 (12.3)	559 (12.8)	622 (14.2)	677 (15.5)
Physical activity^2^					
Moderately inactive	1239 (28.4)	1283 (29.4)	1276 (29.2)	1307 (29.9)	1240 (28.4)
Moderately active	1036 (23.7)	1022 (23.4)	1037 (23.7)	995 (22.8)	991 (22.7)
Active	878 (20.1)	857 (19.6)	824 (18.9)	786 (18.0)	797 (18.3)
Smoking status^2^					
Current	474 (10.9)	446 (10.2)	444 (10.2)	508 (11.6)	698 (16.0)
Former	1860 (42.6)	1833 (42.0)	1853 (42.4)	1708 (39.1)	1637 (37.5)
Hormone replacement therapy (females only)^2^	578 (13.2)	524 (12.0)	496 (11.4)	512 (11.7)	423 (9.7)
Waist circumference (cm)	88.0 ± 12.4	87.8 ± 12.2	87.9 ± 12.2	87.4 ± 11.9	87.3 ± 12.5
BMI (kg/m^2^)	26.7 ± 4.1	26.3 ± 3.8	26.3 ± 3.8	26.1 ± 3.8	25.9 ± 3.8
Hypertension^2^	2070 (47.4)	2019 (46.2)	2002 (45.8)	2027 (46.4)	1991 (45.6)
Hypercholesterolemia^2^	488 (11.2)	333 (7.6)	272 (6.2)	248 (5.7)	159 (3.6)
Diabetes mellitus^2^	90 (2.1)	79 (1.8)	74 (1.7)	88 (2.0)	77 (1.8)
Cancer^2^^,^^3^	269 (6.2)	213 (4.9)	227 (5.2)	235 (5.4)	242 (5.5)
Total energy (kcal)	2017.6 ± 610.5	2118.8 ± 601.4	2075.5 ± 569.3	2021.7 ± 555.2	1988.2 ± 560.7
Alcohol (g)	9.4 ± 14.4	9.5 ± 13.8	8.8 ± 12.7	8.3 ± 12.0	7.5 ± 11.3
Dietary cholesterol (mg)	250.4 ± 112.5	279.4 ± 118.1	279.5 ± 110.5	279.9 ± 109.2	296.6 ± 117.7
Fiber (g)	20.0 ± 7.0	19.5 ± 6.4	19.0 ± 6.3	18.0 ± 5.9	16.4 ± 5.7
Protein (en%)	17.0 ± 3.5	16.4 ± 3.2	16.6 ± 3.0	16.8 ± 3.0	16.6 ± 2.9
Carbohydrate (en%)	52.6 ± 7.2	51.2 ± 6.5	50.6 ± 6.3	49.8 ± 6.0	47.7 ± 6.1
Total fat (en%)	30.5 ± 6.3	32.5 ± 5.7	33.1 ± 5.4	33.8 ± 5.3	36.2 ± 5.2
PUFA (en%)	6.5 ± 2.2	6.4 ± 2.1	6.3 ± 2.0	6.1 ± 1.9	5.6 ± 1.8
MUFA (en%)	10.8 ± 2.6	11.3 ± 2.4	11.4 ± 2.2	11.4 ± 2.2	11.9 ± 2.2
*Trans*-fatty acids (en%)	1.0 ± 0.5	1.1 ± 0.5	1.1 ± 0.4	1.1 ± 0.4	1.2 ± 0.4
Total SFA (en%)	10.5 ± 3.0	11.9 ± 2.8	12.5 ± 2.6	13.4 ± 2.7	15.5 ± 3.0
SFA from food source (en%)					
Dairy	1.0 ± 0.5	2.2 ± 0.3	3.0 ± 0.2	4.0 ± 0.3	6.2 ± 1.6
Meat	1.8 ± 1.2	1.8 ± 1.1	1.8 ± 1.1	1.8 ± 1.1	1.8 ± 1.1
Fish and fish products	0.4 ± 0.3	0.3 ± 0.3	0.3 ± 0.2	0.3 ± 0.2	0.3 ± 0.2
Cereal and cereal products	2.3 ± 1.5	2.4 ± 1.4	2.3 ± 1.3	2.2 ± 1.3	2.1 ± 1.2
Potatoes	0.4 ± 0.4	0.4 ± 0.4	0.4 ± 0.3	0.4 ± 0.4	0.4 ± 0.4
Eggs and egg dishes	0.3 ± 0.4	0.4 ± 0.4	0.4 ± 0.4	0.4 ± 0.4	0.4 ± 0.4
Soups and sauces	0.3 ± 0.4	0.3 ± 0.4	0.3 ± 0.3	0.3 ± 0.3	0.3 ± 0.3
Fats and oils	2.1 ± 2.1	2.3 ± 2.1	2.3 ± 2.0	2.4 ± 2.2	2.7 ± 2.3
Sugars preserves and snacks	1.3 ± 1.2	1.4 ± 1.1	1.3 ± 1.0	1.2 ± 1.0	1.1 ± 1.0
Vegetables	0.2 ± 0.1	0.2 ± 0.1	0.2 ± 0.1	0.2 ± 0.1	0.2 ± 0.1
Dairy products (g)	357.2 ± 190.3	377.3 ± 157.6	419.5 ± 159.3	463.0 ± 165.1	522.0 ± 172.6
Meat products (g)	96.7 ± 53.3	99.7 ± 52.7	96.1 ± 49.1	90.1 ± 46.6	85.4 ± 48.2
Fruit and vegetables (g)	507.6 ± 272.2	484.9 ± 253.5	471.7 ± 238.3	446.3 ± 221.3	406.2 ± 219.4

Abbreviations: EPIC, European Prospective Investigation into Cancer and Nutrition; Q, quintile.

1 Data are in means ± SD for continuous variables or in column percentages (%) for categorical variables.

2 Reference categories not shown for succinctness—sex: male; educational level: no; physical activity status: inactive; smoking status: never; hypertension: no; hypercholesterolemia: no; diabetes mellitus: no; cancer: no; hormone replacement therapy: no.

3 Total missing values, *n* (%): 1 (0.02).

Higher SFA intake from total meat was associated with a lower likelihood of being a female and lower likelihood of having a higher educational degree (Supplemental Table 3). Participants with higher compared with lower meat SFA intake generally had less favorable cardiometabolic risk profile and lifestyle behaviors, including higher waist circumference and BMI, higher likelihood of being a current smoker, and higher intake of alcohol. Furthermore, higher meat SFA intake was associated with higher intake of dietary cholesterol, protein, higher intake of total fat, MUFA, *trans*-fatty acids and total SFA and lower intake of dairy, fruit and vegetables, fiber, and carbohydrate. Baseline characteristics across quantiles of SFA intake from subtypes of meat and dairy are presented in Supplemental Tables 4–9.

### Associations with incident CVD

**FIGURE 1:**
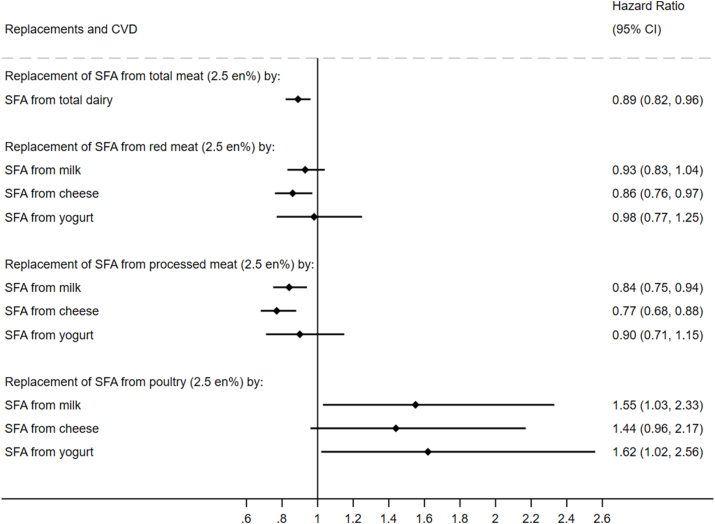
Modeled replacement of SFA from total, red, processed, or poultry meat (2.5% of total energy) by the equivalent from total and types of dairy in relation to incident CVD: the EPIC-Norfolk Study (*N* = 21,841). Multivariable-adjusted Cox model included age, sex, educational level, physical activity, smoking status, alcohol intake, fiber intake, fruit and vegetables, hormone replacement therapy (females only), dietary cholesterol, *trans*-fatty acids, protein, carbohydrate, PUFA, MUFA, SFA from other foods, BMI, waist circumference, baseline hypertension, baseline hypercholesterolemia, and baseline diabetes mellitus. No. of CVD cases: 5902 over person-years of 395,854.5. CVD, cardiovascular disease; en%, percentage of total energy intake; EPIC, European Prospective Investigation into Cancer and Nutrition.

**FIGURE 2:**
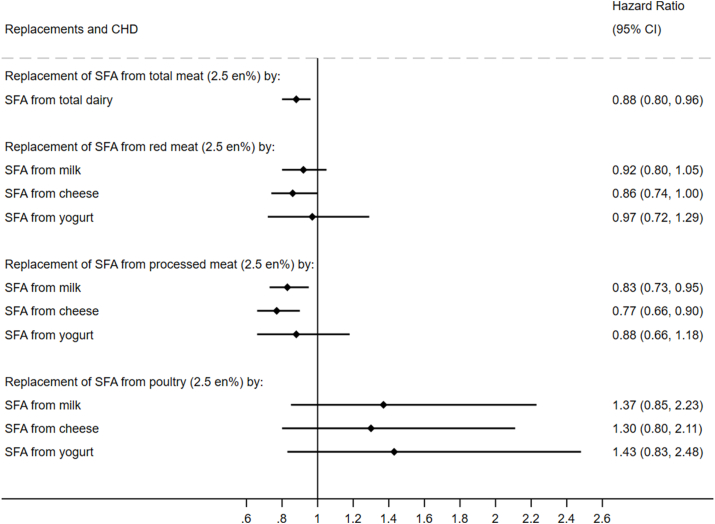
Modeled replacement of SFA from total, red, processed, or poultry meat (2.5% of total energy) by the equivalent from total and types of dairy in relation to incident CAD: the EPIC-Norfolk Study (*N* = 21,841). Multivariable-adjusted Cox model included age, sex, educational level, physical activity, smoking status, alcohol intake, fiber intake, fruit and vegetables, hormone replacement therapy (females only), dietary cholesterol, *trans*-fatty acids, protein, carbohydrate, PUFA, MUFA, SFA from other foods, BMI, waist circumference, baseline hypertension, baseline hypercholesterolemia, and baseline diabetes mellitus. No. of CAD cases: 4215 over person years of 401,846.7. CAD, coronary artery disease; en%, percentage of total energy intake; EPIC, European Prospective Investigation into Cancer and Nutrition.

**FIGURE 3:**
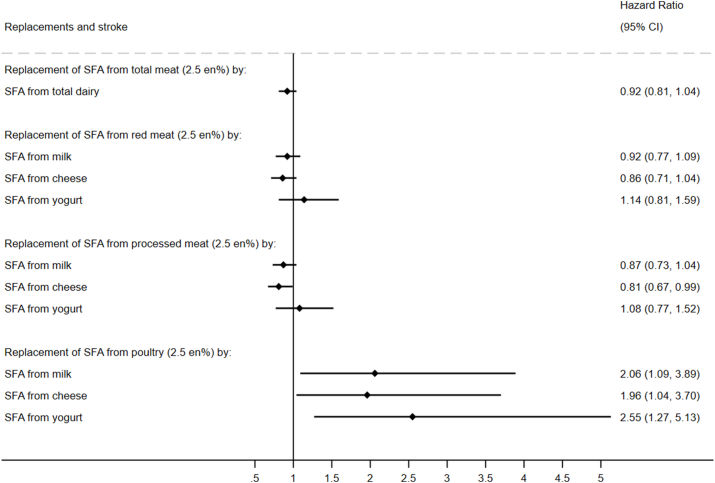
Modeled replacement of SFA from total, red, processed, or poultry meat (2.5% of total energy) by the equivalent from total and types of dairy in relation to incident stroke: the EPIC-Norfolk Study (*N* = 21,841). Multivariable-adjusted Cox model included age, sex, educational level, physical activity, smoking status, alcohol intake, fiber intake, fruit and vegetables, hormone replacement therapy (females only), dietary cholesterol, *trans*-fatty acids, protein, carbohydrate, PUFA, MUFA, SFA from other foods, BMI, waist circumference, baseline hypertension, baseline hypercholesterolemia, and baseline diabetes mellitus. No. of stroke cases: 2544 over person years of 419,904.3. en%, percentage of total energy intake; EPIC, European Prospective Investigation into Cancer and Nutrition.

During a median follow-up of 21.0 y and between 1993 and 2018 (395,854.5 person-years for incident CVD; 401,846.7 person-years for incident CAD; 419,904.3 person-years for incident stroke; 423,746.3 person-years for incident ischemic stroke; and 426,640.6 person-years for incident hemorrhagic stroke), we identified 5902 cases (27.0%) of incident CVD; 4215 cases (19.2%) of incident CAD; 2544 cases (11.6%) of incident stroke; 1113 cases (5.1%) of incident ischemic stroke, and 449 cases (2.1%) of incident hemorrhagic stroke, in our study population of 21,841. For each CVD outcome of interest, results of the multivariable-adjusted models are presented in forest plots (FIGURE 1, FIGURE 2, FIGURE 3, Supplemental Figures 2 and 3). Results of the unadjusted and other adjusted models are presented in Supplemental Table 10.

Multivariable-adjusted models, including sociodemographic, lifestyle, cardiometabolic, and dietary factors, showed that the replacement of 2.5 en% of SFA from total meat with the same amount of energy of SFA from total dairy was associated with a 11% lower incidence of CVD (HR: 0.89; 95% CI: 0.82, 0.96) (Figure 1). Analyses of individual meat and dairy types revealed lower incident CVD with replacement of SFA from red meat by cheese (HR: 0.86; 95% CI: 0.76, 0.97). Similarly, replacing SFA from processed meat by milk (HR: 0.84; 95% CI: 0.75, 0.94) or cheese (HR: 0.77; 95% CI: 0.68, 0.88) was associated with significantly lower CVD incidence. On the contrary, the replacement of SFA from poultry by milk or yogurt was associated with elevated incidence of CVD, with HR of 1.55 (95% CI: 1.03, 2.33) and 1.62 (95% CI: 1.02, 2.56), respectively (Figure 1).

### Associations with incident CAD

Similar findings as for CVD were found for CAD. Replacing 2.5 en% of SFA from total meat by the equivalent from total dairy was associated with a lower incident CAD of 12% (HR: 0.88; 95% CI: 0.80, 0.96) in the multivariable-adjusted models (Figure 2). Different types of meat and dairy products were not associated with CAD risk, except for the replacement of SFA from processed meat by milk (HR: 0.83; 95% CI: 0.73, 0.95) and cheese (HR: 0.77; 95% CI: 0.66, 0.90) (Figure 2).

### Associations with incident stroke

For stroke, a lower incidence was observed for the replacement of SFA from processed meat by cheese (HR: 0.81; 95% CI: 0.67, 0.99) (Figure 3). In contrast, replacement of SFA from poultry by milk (HR: 2.06; 95% CI: 1.09, 3.89), cheese (HR: 1.96; 95% CI: 1.04, 3.70), or yogurt (HR: 2.55; 95% CI: 1.27, 5.13) was associated with an estimated higher stroke incidence significantly (Figure 3). For incident ischemic and hemorrhagic stroke, no significant associations were detected or total and individual meat and dairy types (Supplemental Figures 2 and 3).

### Association analyses without replacements

A higher SFA intake from total and processed meat was associated with higher incidence of CVD (HR: 1.18; 95% CI: 1.05, 1.27) and CAD (HR: 1.19; 95% CI: 1.04, 1.37) (Supplemental Table 11). Although red meat (HR: 1.00; 95% CI: 0.82, 1.20) and processed meat (HR: 1.11; 95% CI: 0.92, 1.34) were not associated with stroke incidence, SFA from poultry was significantly associated with a lower stroke incidence (HR: 0.49; 95% CI: 0.25, 0.96). For dairy foods, a higher intake of SFA from cheese was consistently associated with significant lower incidence of CVD (HR: 0.91; 95% CI: 0.84, 0.99) (Supplemental Table 11).

### Sensitivity and secondary analyses

Analyses using age as underlying time scale did not alter the results (Supplemental Table 12). Moreover, results did not change when excluding incident cases in the first 2 y of follow-up or when participants with self-reported baseline diabetes or cancer were excluded from analysis (Supplemental Table 12), except for the replacement of SFA from poultry by milk and yogurt with overall CVD. The association became nonsignificant when these participants were excluded from analyses. No evidence was found for an interaction by sex and age for the associations with all outcomes of interest (Supplemental Table 13). Similarly, no evidence was observed for interaction by baseline comorbidity (*P*-interaction > 0.05), except for the association with incident stroke (*P*-interaction = 0.0492). In a sensitivity analysis restricting the study population to those with a baseline comorbidity, a borderline significant inverse association with stroke incidence was observed with replacement of SFA from total meat by total dairy (HR: 0.86; 95% CI: 0.74, 1.00), whereas among participants with no or unknown comorbidity, no significant association was found (HR: 1.08; 95% CI: 0.87, 1.34) (Supplemental Table 13).

## Discussion

To our knowledge, this is the first analysis of the associations of replacing SFA from different meat products by dairy products with incident total and subtypes of CVD in the UK population. In this cohort of 21,841 UK adults, isoenergetic replacement of SFA from total meat by total dairy (2.5 en%) was significantly associated with 11% and 12% lower incidence of CVD and CAD, respectively, in line with previous prospective cohort studies [4,5].The EPIC-CVD cohort revealed that replacing 100 kcal/d (418 kJ/d) from red and processed meat with 100 kcal/d from yogurt or cheese, but not milk, was significantly associated with lower risk of CAD [20]. This may be due to a lower SFA intake and/or other beneficial components of the matrices of yogurt or cheese (e.g., bioactive peptides and short-chain fatty acids). A large prospective case–cohort study showed that the replacement of 5 en% of SFA from red meat by other (nonspecified) foods was associated with 30% lower CAD incidence [21]. However, the replacement of 5 en% of SFA from fermented dairy products by other food sources was associated with 20% higher CAD incidence, suggesting the importance of fermented dairy foods (yogurt and cheese), as moderators of CAD risk [21]. For CAD, our findings generally support previous results [20,21]. However, our findings did not suggest significantly lower risk with replacement of SFA from either red or processed meat by SFA from yogurt. An explanation for this could be the low SFA intake from yogurt, resulting in less precise estimates and lower statistical power. Moreover, the nonsignificant findings could be due to the sugars and flavors, which were commonly added to yogurt products in the United Kingdom in 1990s.

To date, no isoenergetic replacement of SFA from total and individual meat products by total and individual dairy products on stroke incidence has been shown. Lower stroke incidence was observed with replacement of SFA from processed meat by cheese although higher stroke incidence resulted from replacement of SFA from poultry by dairy products. Other studies have showed the relationship between red meat intake and stroke incidence has been inconsistent, with studies showing nonsignificant [22,23] or positive associations [24,25], whereas processed meat has been consistently associated with higher stroke incidence [22,25]. Poultry meat has not been shown to be significantly associated with stroke incidence [26]. Thus, the associations between meat intake and stroke may differ, depending on the type of meat, which agrees with our study findings. It also adds evidence to the proposal that red meat and processed meat should have separate dietary guidelines [5]. The general neutrality of poultry meat and the neutrality/reduced risk of dairy foods and CVD risk are not in line with the findings of this study. This shows that replacement of poultry SFA by dairy product SFA were associated with a higher incidence of CVD and stroke. This may be a function of the relatively low contribution to SFA intake from the poultry meat, leading to imprecise estimates of risk, as reflected in the wider CI seen. More replication studies are needed, to add evidence on the effect estimates.

Lower CVD risk following replacing SFA from total meat by total dairy could be due to the different proportions of individual SFA in meat and dairy products and their possible differential impact on CVD risk, as suggested previously [4,5], although the evidence is limited in this regard. Higher circulating concentrations of odd-chain dairy fat biomarkers 15:0 and 17:0 has been shown to be associated with lower CVD risk, but any underlying mechanism remains unclear and may simply be a biomarker of dairy intake [27]. Another explanation for the observed benefit of dairy products, in comparison with meat products, could be the impact of other constituents within the meat (e.g., sodium, preservatives, and nitrates) and dairy matrices (e.g., protein and calcium) and other constituents (e.g., bacteria and milk fat globule membrane), which may modulate the impact of SFA on CVD risk [28].

Strengths of this study include the prospective design with a long follow-up period, the large sample size, and the relatively high number of incident cases of CVD. In addition, a comprehensive set of confounders were adjusted for in the analyses and the robustness of the results were assessed and confirmed by multiple sensitivity analyses. Evaluation of replacing SFA from different types of meat by SFA different types of dairy products provides novel insights into the associations of SFA from specific types of meat and dairy with risk of overall and subtypes of CVD.

This study also has several limitations. First, results from the replacement models should be interpreted with caution, as these were obtained from statistical models using dietary intakes in the study population and were not actual replacements. Therefore, causal relations cannot be inferred. Second, the correlations between the estimates of meat-specific and dairy-specific SFA with the corresponding food sources were moderate to high, which makes it challenging to separate the effects of SFA from their food sources. Third, we use dietary data, collected from FFQs. Misclassification of participants might bias our findings in unknown direction, as the estimation of an participants’ (average) food consumption relied on their memory and ability to recall and estimate their food intake. Fourth, we were unable to investigate the associations of SFA from unprocessed compared with processed poultry in our replacement models because the FFQ did not contain this information. Future studies investigating the associations of SFA by unprocessed and processed poultry with CVD are, therefore, needed, due to their potential differential impact on health [29]. Fifth, dietary and covariate data were collected from one measurement at baseline, which precluded correction for within-person changes in many characteristics. Sixth, we used dietary data, collected in 1990s in Norfolk, England, which may not reflect current dietary patterns in the United Kingdom or elsewhere. Finally, our study population was predominantly White, which limits the generalizability of our results to other ethnic/racial populations.

In conclusion, replacement of SFA from meat products, and especially processed meat, by dairy products may lower incidence of CVD and CAD. Our findings also add to the evidence that different types of meat (notably poultry compared with red) should be considered separately in future analyses and in the current dietary recommendations. Replication and intervention studies are needed to confirm our findings, ideally in other populations, with diets that reflect habitual dietary intake. Future research should also focus on food replacements of different types of red meat (unprocessed and processed) by different types of poultry (unprocessed and processed) to further inform more specific food-based dietary recommendation for CVD prevention.

## Author contributions

The authors’ responsibilities were as follows – YDV, SSS-M, FI, DIG, JAL: designed the research; YDV: analyzed the data; SSS-M, FI, DIG, JAL: provided supervision; YDV: wrote the first draft of the paper; YDV, SSS-M, FI, DIG, JAL: reviewed, edited, wrote the paper, interpreted the data, and critically revised the paper for important intellectual content; and all authors have read and approved the final manuscript.

## Conflict of interest

YDV has received funding for her PhD studentship from the Rank Prize Funds, Dutch Dairy Association (NZO), and the Danish Dairy Research Foundation. SSS-M has received an unrestricted grant from the Dutch Dairy Association. FI is an Editorial Board Member for the *American Journal of Clinical Nutrition* and played no role in the Journal’s evaluation of the manuscript. FI has received funds from the UK Medical Research Council (MC_UU_12015/5, MC_UU_00006/3). DIG has received travel expenses and honoraria in connection with lectures and meetings from the Dairy Council (now Dairy UK), Dutch Dairy Association, European Dairy Association, and the International Dairy Federation. He has also been a consultant to the Estonian Biocompetence Centre of Healthy Dairy Products (BioCC) and to the Dairy Council on fats in dairy products and cardiometabolic diseases. JAL is Deputy Chair of the UK Governments Scientific Advisory Committee on Nutrition (SACN).

## Funding

The EPIC-Norfolk study (https://doi.org/10.22025/2019.10.105.00004) has received funding from the Medical Research Council (MR/N003284/1, MC_UU_12015/1, MC_UU_00006/1) and Cancer Research UK (C864/A14136). Funding for PhD studentship was provided to YDV by the Rank Prize Funds, Dutch Dairy Association (NZO), and the Danish Dairy Research Foundation. These organizations were not involved in the design, collection, analysis, or interpretation of the data, nor were they involved in the writing of the manuscript or the decision to submit it for publication.

## Data availability

Data described in this manuscript, code book, and analytic code will be made available on request pending approval by the EPIC-Norfolk Management Committee (E-mail: epic-norfolk@mrc-epid.cam.ac.uk).
