# Supplementary material for: Replacement of Saturated Fatty Acids from Meat by Dairy Sources in Relation to Incident Cardiovascular Disease: The European Prospective Investigation into Cancer and Nutrition (EPIC)-Norfolk Study
Source: Am J Clin Nutr. 2024 Apr 11;119(6):1495–503. doi: 10.1016/j.ajcnut.2024.04.007 (PMC11196860; doi:10.1016/j.ajcnut.2024.04.007)
Supplement: Multimedia component 1 [file mmc1.docx]

**Supplementary materials**

**Table of contents**

[**SUPPLEMENTARY TABLE 1** Food items included in the meat and dairy groups, as sources of saturated fatty acids, EPIC-Norfolk Study^1^ 3](#_Toc163048910)

[**SUPPLEMENTARY TABLE 2** Correlation coefficients and 95% confidence intervals between estimates of total, meat- and dairy-specific saturated fatty acids (expressed as percentage of total energy intake) at baseline: 1993-1997, EPIC-Norfolk Study (n=21,841)^1^ 4](#_Toc163048911)

[**SUPPLEMENTARY TABLE 3** Baseline characteristics across quintiles of saturated fatty acids intake from total meat (expressed as percentage of total energy intake): 1993-1997, EPIC-Norfolk Study (n=21,841)^1^ 5](#_Toc163048912)

[**SUPPLEMENTARY TABLE 4** Baseline characteristics across quintiles of saturated fatty acids intake from milk (expressed as percentage of total energy intake): 1993-1997, EPIC-Norfolk Study (n=21,841)^1^ 7](#_Toc163048913)

[**SUPPLEMENTARY TABLE 5** Baseline characteristics across tertiles of saturated fatty acids intake from yogurt (expressed as percentage of total energy intake): 1993-1997, EPIC-Norfolk Study (n=21,841)^1^ 9](#_Toc163048914)

[**SUPPLEMENTARY TABLE 6** Baseline characteristics across quintiles of saturated fatty acids intake from cheese (expressed as percentage of total energy intake): 1993-1997, EPIC-Norfolk Study (n=21,841)^1^ 11](#_Toc163048915)

[**SUPPLEMENTARY TABLE 7** Baseline characteristics across quintiles of saturated fatty acids intake from red meat (expressed as percentage of total energy intake): 1993-1997, EPIC-Norfolk Study (n=21,841)^1^ 13](#_Toc163048916)

[**SUPPLEMENTARY TABLE 8** Baseline characteristics across quintiles of saturated fatty acids intake from processed meat (expressed as percentage of total energy intake): 1993-1997, EPIC-Norfolk Study (n=21,841)^1^ 15](#_Toc163048917)

[**SUPPLEMENTARY TABLE 9** Baseline characteristics across quintiles of saturated fatty acids intake from poultry (expressed as percentage of total energy intake): 1993-1997, EPIC-Norfolk Study (n=21,841)^1^ 17](#_Toc163048918)

[**SUPPLEMENTARY TABLE 10** Hazard ratios and 95% confidence intervals for risk of overall and subtypes of cardiovascular disease associated with the Modeled replacement of saturated fatty acids from total and types of meat by total and types of dairy in the EPIC-Norfolk Study (n=21,841)^1^ 20](#_Toc163048919)

[**SUPPLEMENTARY TABLE 11** Hazard ratios and 95% confidence intervals for the risk of overall and subtypes of cardiovascular disease associated with saturated fatty acid intakes from total and types of meat by total and types of dairy in the EPIC-Norfolk Study (n=21,841)^1^ 23](#_Toc163048920)

[**SUPPLEMENTARY TABLE 12** Hazard ratios and 95% confidence intervals for risk of cardiovascular disease associated with the Modeled replacement of saturated fatty acids from total and types of meat by total and types of dairy in the EPIC-Norfolk Study (n=21,841): Sensitivity analyses^1^ 24](#_Toc163048921)

[**SUPPLEMENTARY TABLE 13** Hazard ratios and 95% confidence intervals for risk of overall and subtypes of cardiovascular disease associated with the Modeled replacement of saturated fatty acids from total meat by total dairy, stratified by potential effect modifiers, in the EPIC-Norfolk Study (n=21,841)^1^ 26](#_Toc163048922)

[**SUPPLEMENTARY FIGURE 1** Flowchart of participant selection for the analyses on the estimated effect of isoenergetic replacement of SFA from meat by SFA from dairy on the incidence of CVD in adults of the EPIC-Norfolk Study^1^ 28](#_Toc163048923)

[**SUPPLEMENTARY FIGURE 2** Modeled replacement of SFA from total, red, processed or poultry meat (2.5% of total energy) by the equivalent from total and types of dairy in relation to incident ischemic stroke: the EPIC-Norfolk Study(n=21,841)^1^ 30](#_Toc163048924)

[**SUPPLEMENTARY FIGURE 3** Modeled replacement of SFA from total, red, processed or poultry meat (2.5% of total energy) by the equivalent from total and types of dairy in relation to incident hemorrhagic stroke: the EPIC-Norfolk Study(n=21,841)^1^ 32](#_Toc163048925)

**SUPPLEMENTARY TABLE 1** Food items included in the meat and dairy groups, as sources of saturated fatty acids, EPIC-Norfolk Study^1^

|  | Food items |
| --- | --- |
| Total meat | Red and processed meat, poultry |
| Red meat (unprocessed and processed) | Beef, beef burgers, pork, lamb, lasagne or moussaka, liver and liver containing products (liver pate and sausages) |
| Processed meat | Bacon, ham, corned beef, spam, luncheon meats, sausages, savoury pies (e.g., meat pie, pork pie, pasties, steak, kidney pie, sausage rolls) |
| Poultry | Chicken or turkey |
| Total dairy | Milk, yogurt, cheese, other dairy products |
| Milk | Full-cream silver milk, skimmed/blue milk, dried milk, semi-skimmed milk, Channel Islands milk, goat’s milk, sheep’s milk, calcium fortified milk, evaporated milk, skimmed milk |
| Yogurt | Low-fat yogurt, full-fat yogurt |
| Cheese | Hard cheese, cottage, or low-fat soft cheese |

^1^EPIC, European Prospective Investigation into Cancer and Nutrition

# **SUPPLEMENTARY TABLE 2** Correlation coefficients and 95% confidence intervals between estimates of total, meat- and dairy-specific saturated fatty acids (expressed as percentage of total energy intake) at baseline: 1993-1997, EPIC-Norfolk Study (n=21,841)^1^

| SFA food sources | Spearman rank correlation coefficient (*r*_s_) between SFA intakes from their food sources | | | | | | | | *r*_s_ between  SFA intake from each food source (% energy) and its food intake (grams/day) |
| --- | --- | --- | --- | --- | --- | --- | --- | --- | --- |
|  | All sources | Total meat | Red meat | Processed meat | Poultry | Total dairy | Milk | Cheese |  |
| All sources | 1.00 |  |  |  |  |  |  |  |  |
| Total meat | 0.26 (0.25, 0.27) | 1.00 |  |  |  |  |  |  | 0.85 (0.85, 0.85) |
| Red meat | 0.24 (0.23, 0.25) | 0.82 (0.82, 0.82) | 1.00 |  |  |  |  |  | 0.85 (0.85, 0.85) |
| Processed meat | 0.30 (0.29, 0.31) | 0.80 (0.80, 0.80) | 0.43 (0.42, 0.44) | 1.00 |  |  |  |  | 0.94 (0.94, 0.94) |
| Poultry | -0.24 (-0.25, -0.23) | 0.19 (0.18, 0.20) | 0.06 (0.05, 0.07) | -0.03 (-0.04, -0.02) | 1.00 |  |  |  | 1.00 |
| Total dairy | 0.53 (0.52, 0.54) | -0.01 (-0.02, 0.00) | 0.03 (0.02, 0.04) | 0.00 (-0.01, 0.01) | -0.15 (-0.16, -0.14) | 1.00 |  |  | 0.45 (0.44, 0.46) |
| Milk | 0.46 (0.45, 0.47) | 0.05 (0.04, 0.06) | 0.07 (0.06, 0.08) | 0.07 (0.06, 0.08) | -0.13 (-0.14, -0.12) | 0.78 (0.77, 0.79) | 1.00 |  | 0.56 (0.55, 0.57) |
| Cheese | 0.17 (0.16, 0.18) | -0.07 (-0.08, -0.06) | -0.04 (-0.05, -0.03) | -0.05 (-0.06, -0.04) | -0.08 (-0.09, -0.07) | 0.44 (0.43, 0.45) | -0.02 (-0.03, -0.01) | 1.00 | 0.90 (0.90, 0.90) |
| Yogurt | -0.21 (-0.22, -0.20) | -0.18 (-0.19, -0.17) | -0.12 (-0.13, -0.11) | -0.21 (-0.22, -0.20) | 0.09 (0.08, 0.10) | 0.05 (0.04, 0.06) | -0.10 (-0.11, -0.09) | 0.08 (0.07, 0.09) | 0.94 (0.94, 0.94) |

EPIC, European Prospective Investigation into Cancer and Nutrition; SFA, saturated fatty acids

# **SUPPLEMENTARY TABLE 3** Baseline characteristics across quintiles of saturated fatty acids intake from total meat (expressed as percentage of total energy intake): 1993-1997, EPIC-Norfolk Study (n=21,841)^1^

|  | Q1  (n=4369) | | Q2  (n=4368) | Q3  (n=4368) | Q4  (n=4368) | Q5  (n=4368) |
| --- | --- | --- | --- | --- | --- | --- |
| Range of SFA from total meat, en% | 0-0.9 | | 0.9-1.4 | 1.4-1.8 | 1.8-2.5 | 2.5-12.3 |
| Age (years) | 58.7 ±9.3 | | 59.2 ±9.1 | 58.7 ±9.1 | 58.4 ±9.2 | 57.7 ±9.2 |
| Sex^2^_,_ female | 2765 (63.3) | | 2665 (61.0) | 2556 (58.5) | 2381 (54.5) | 1950 (44.6) |
| Educational level^2^ |  | |  |  |  |  |
| Ordinary-level | 440 (10.1) | | 428 (9.8) | 501 (11.5) | 469 (10.7) | 461 (10.6) |
| Advanced-level | 1770 (40.5) | | 1787 (40.9) | 1708 (39.1) | 1728 (39.6) | 1807 (41.4) |
| Degree | 841 (19.3) | | 595 (13.6) | 528 (12.1) | 479 (11.0) | 459 (10.5) |
| Physical activity^2^ |  | |  |  |  |  |
| Moderately inactive | | 1335 (30.6) | 1304 (29.9) | 1272 (29.1) | 1239 (28.4) | 1195 (27.4) |
| Moderately active | 1034 (23.7) | | 1023 (23.4) | 1025 (23.5) | 1017 (23.3) | 982 (22.5) |
| Active | 854 (19.6) | | 819 (18.8) | 786 (18.0) | 810 (18.5) | 873 (20.0) |
| Smoking status^2^ |  | |  |  |  |  |
| Current | 401 (9.2) | | 407 (9.3) | 451 (10.3) | 509 (11.7) | 802 (18.4) |
| Former | 1700 (38.9) | | 1749 (40.0) | 1804 (41.3) | 1827 (41.8) | 1811 (41.5) |
| Hormone replacement therapy (females only)^2^ | 580 (13.3) | | 535 (12.3) | 500 (11.5) | 500 (11.5) | 418 (9.6) |
| Waist circumference (cm) | 84.9 ±11.7 | | 86.4 ±11.7 | 87.5 ±11.9 | 88.9 ±12.5 | 90.8 ±12.4 |
| BMI (kg/m^2^) | 25.4 ±3.7 | | 26.0 ±3.7 | 26.3 ±3.7 | 26.6 ±3.9 | 26.9 ±4.0 |
| Hypertension^2^ | 1927 (44.1) | | 2044 (46.8) | 2090 (47.9) | 2032 (46.5) | 2016 (46.2) |
| Hypercholesterolemia^2^ | 358 (8.2) | | 321 (7.4) | 325 (7.4) | 263 (6.0) | 233 (5.3) |
| Diabetes mellitus^2^ | 72 (1.7) | | 83 (1.9) | 72 (1.7) | 73 (1.7) | 108 (2.5) |
| Cancer^2,3^ | 255 (5.8) | | 249 (5.7) | 235 (5.4) | 229 (5.2) | 218 (5.0) |
| Total energy (kcal) | 2068.6 ±604.5 | | 2084.3 ±574.8 | 2045.3 ±565.2 | 1999.0 ±572.5 | 2024.5 ±586.8 |
| Alcohol (g) | 8.0 ±12.1 | | 8.2 ±12.5 | 8.5 ±12.8 | 8.7 ±12.5 | 10.0 ±14.4 |
| Dietary cholesterol (mg) | 228.9 ±108.0 | | 264.9 ±104.9 | 278.8 ±106.7 | 288.4 ±106.1 | 324.6 ±124.6 |
| Fiber (g) | 21.2 ±7.3 | | 19.5 ±6.3 | 18.5 ±5.9 | 17.4 ±5.7 | 16.2 ±5.6 |
| Protein (en %) | 15.2 ±2.8 | | 16.2 ±2.9 | 16.7 ±2.9 | 17.3 ±3.0 | 18.0 ±3.2 |
| Carbohydrate (en %) | 54.2 ±6.6 | | 52.3 ±5.9 | 50.8 ±5.7 | 49.1 ±5.5 | 45.5 ±5.9 |
| Total fat (en %) | 31.4 ±6.4 | | 32.1 ±5.8 | 32.8 ±5.4 | 33.8 ±5.4 | 36.0 ±5.3 |
| PUFA (en %) | 6.5 ±2.3 | | 6.3 ±2.1 | 6.1 ±1.9 | 6.1 ±1.9 | 6.1 ±1.8 |
| MUFA (en %) | 10.4 ±2.5 | | 10.8 ±2.3 | 11.2 ±2.1 | 11.6 ±2.1 | 12.7 ±2.1 |
| Trans fatty acids (en %) | 1.0 ±0.5 | | 1.1 ±0.5 | 1.1 ±0.5 | 1.1 ±0.4 | 1.2 ±0.4 |
| Total SFA (en %) | 11.7 ±3.5 | | 12.2 ±3.2 | 12.7 ±3.1 | 13.1 ±3.1 | 14.1 ±3.0 |
| SFA from food source  (en %) |  | |  |  |  |  |
| Dairy | 3.4 ±2.1 | | 3.3 ±1.9 | 3.3 ±1.9 | 3.3 ±1.8 | 3.2 ±1.9 |
| Meat | 0.6 ±0.3 | | 1.2 ±0.1 | 1.6 ±0.1 | 2.2 ±0.2 | 3.5 ±1.0 |
| Fish and fish products | 0.3 ±0.3 | | 0.3 ±0.3 | 0.3 ±0.2 | 0.3 ±0.2 | 0.3 ±0.2 |
| Cereal and cereal products | 2.4 ±1.5 | | 2.4 ±1.4 | 2.4 ±1.3 | 2.2 ±1.3 | 2.0 ±1.2 |
| Potatoes | 0.3 ±0.3 | | 0.3 ±0.3 | 0.4 ±0.3 | 0.4 ±0.4 | 0.5 ±0.4 |
| Eggs and egg dishes | 0.4 ±0.4 | | 0.3 ±0.3 | 0.3 ±0.4 | 0.4 ±0.3 | 0.4 ±0.4 |
| Soups and sauces | 0.2 ±0.3 | | 0.3 ±0.4 | 0.3 ±0.4 | 0.3 ±0.3 | 0.3 ±0.3 |
| Fats and oils | 2.5 ±2.2 | | 2.4 ±2.2 | 2.3 ±2.1 | 2.3 ±2.1 | 2.3 ±2.1 |
| Sugars preserves and snacks | 1.2 ±1.2 | | 1.3 ±1.1 | 1.3 ±1.1 | 1.3 ±1.0 | 1.2 ±1.0 |
| Vegetables | 0.2 ±0.2 | | 0.2 ±0.1 | 0.2 ±0.1 | 0.2 ±0.1 | 0.2 ±0.1 |
| Dairy products (g) | 457.0 ±188.5 | | 445.7 ±175.1 | 430.6 ±175.9 | 411.9 ±174.2 | 393.7 ±176.4 |
| Meat products (g) | 41.3 ±27.0 | | 77.5 ±25.4 | 94.3 ±30.5 | 109.2 ±34.1 | 145.7 ±57.0 |
| Fruit and vegetables (g) | 537.2 ±287.2 | | 486.1 ±243.7 | 461.2 ±231.6 | 432.5 ±222.0 | 399.6 ±206.3 |

^1^Data are in means ±SD for continuous variables or in column percentages (%) for categorical variables. En%, percentage of total energy intake; EPIC, European Prospective Investigation into Cancer and Nutrition; Q, quintile; SFA, saturated fatty acids. ^2^Reference categories not shown for succinctness: sex: male; educational level: no; physical activity status: inactive; smoking status: never; hypertension: no; hypercholesterolemia: no; diabetes mellitus: no; cancer: no; hormone replacement therapy: no. ^3^Total missing values n (%): 1 (0.02)

# **SUPPLEMENTARY TABLE 4** Baseline characteristics across quintiles of saturated fatty acids intake from milk (expressed as percentage of total energy intake): 1993-1997, EPIC-Norfolk Study (n=21,841)^1^

|  | Q1  (n=4369) | | Q2  (n=4368) | Q3  (n=4368) | Q4  (n=4368) | Q5  (n=4368) |
| --- | --- | --- | --- | --- | --- | --- |
| Range of SFA from milk, en% | 0-0.1 | | 0.1-1.2 | 1.2-1.8 | 1.8-2.7 | 2.7-14.9 |
| Age (years) | 57.5 ±8.8 | | 57.8 ±9.1 | 58.9 ±9.3 | 58.6 ±9.2 | 59.8 ±9.4 |
| Sex^2^, female | 2,662 (60.9) | | 2,479 (56.8) | 2,443 (55.9) | 2,461 (56.3) | 2,272 (52.0) |
| Educational level^2^ |  | |  |  |  |  |
| Ordinary-level | 450 (10.3) | | 464 (10.6) | 473 (10.8) | 460 (10.5) | 452 (10.4) |
| Advanced-level | 1,768 (40.5) | | 1,792 (41.0) | 1,768 (40.5) | 1,793 (41.1) | 1,679 (38.4) |
| Degree | 651 (14.9) | | 603 (13.8) | 558 (12.8) | 555 (12.7) | 535 (12.3) |
| Physical activity^2^ |  | |  |  |  |  |
| Moderately inactive | | 1,314 (30.1) | 1,247 (28.6) | 1,317 (30.2) | 1,303 (29.8) | 1,164 (26.7) |
| Moderately active | 1,061 (24.3) | | 1,028 (23.5) | 1,036 (23.7) | 985 (22.6) | 971 (22.2) |
| Active | 848 (19.4) | | 864 (19.8) | 816 (18.7) | 822 (18.8) | 792 (18.1) |
| Smoking status^2^ |  | |  |  |  |  |
| Current | 421 (9.6) | | 436 (10.0) | 447 (10.2) | 496 (11.4) | 770 (17.6) |
| Former | 1,872 (42.9) | | 1,848 (42.3) | 1,832 (41.9) | 1,709 (39.1) | 1,630 (37.3) |
| Hormone replacement therapy (females only)^2^ | 611 (14.0) | | 549 (12.6) | 505 (11.6) | 484 (11.1) | 384 (8.8) |
| Waist circumference (cm) | 87.5 ±12.3 | | 87.6 ±12.2 | 87.9 ±12.1 | 87.6 ±12.3 | 87.9 ±12.2 |
| BMI (kg/m^2^) | 26.6 ±4.0 | | 26.3 ±3.9 | 26.2 ±3.7 | 26.2 ±3.9 | 26.0 ±3.7 |
| Hypertension^2^ | 1,995 (45.7) | | 1,958 (44.8) | 2,053 (47.0) | 2,045 (46.8) | 2,058 (47.1) |
| Hypercholesterolemia^2^ | 457 (10.5) | | 342 (7.8) | 266 (6.1) | 246 (5.6) | 189 (4.3) |
| Diabetes mellitus^2^ | 77 (1.8) | | 77 (1.8) | 79 (1.8) | 92 (2.1) | 83 (1.9) |
| Cancer^2,3^ | 260 (6.0) | | 225 (5.2) | 232 (5.3) | 240 (5.5) | 229 (5.2) |
| Total energy (kcal) | 1,962.7 ±560.5 | | 2,110.5 ±654.0 | 2,139.7 ±523.8 | 1,998.0 ±565.6 | 2,010.8 ±576.6 |
| Alcohol (g) | 9.9 ±13.9 | | 10.1 ±14.3 | 9.0 ±12.8 | 7.4 ±11.4 | 7.0 ±11.5 |
| Dietary cholesterol (mg) | 242.1 ±103.7 | | 272.0 ±121.1 | 288.3 ±105.4 | 277.7 ±113.2 | 305.6 ±119.0 |
| Fiber (g) | 19.9 ±6.9 | | 19.5 ±6.8 | 19.3 ±6.0 | 18.0 ±6.0 | 16.2 ±5.6 |
| Protein (en %) | 17.1 ±3.3 | | 16.5 ±3.5 | 16.3 ±2.7 | 17.0 ±3.0 | 16.5 ±3.0 |
| Carbohydrate (en %) | 51.7 ±7.1 | | 51.0 ±6.9 | 50.5 ±6.2 | 50.4 ±6.1 | 48.2 ±6.2 |
| Total fat (en %) | 30.9 ±6.0 | | 32.4 ±6.2 | 33.5 ±5.2 | 33.3 ±5.3 | 36.0 ±5.4 |
| PUFA (en %) | 6.5 ±2.1 | | 6.5 ±2.1 | 6.4 ±2.0 | 6.1 ±1.9 | 5.6 ±1.8 |
| MUFA (en %) | 10.8 ±2.5 | | 11.2 ±2.6 | 11.5 ±2.2 | 11.3 ±2.2 | 12.0 ±2.2 |
| Trans fatty acids (en %) | 1.0 ±0.5 | | 1.1 ±0.5 | 1.1 ±0.4 | 1.1 ±0.4 | 1.2 ±0.4 |
| Total SFA (en %) | 10.8 ±2.9 | | 11.9 ±3.1 | 12.7 ±2.6 | 13.0 ±2.8 | 15.3 ±3.1 |
| SFA from food source  (en %) |  | |  |  |  |  |
| Milk | 0.0 ±0.0 | | 0.7 ±0.4 | 1.5 ±0.2 | 2.2 ±0.3 | 4.2 ±1.5 |
| Yogurt | 0.2 ±0.3 | | 0.1 ±0.3 | 0.1 ±0.3 | 0.1 ±0.3 | 0.1 ±0.3 |
| Cheese | 1.2 ±1.0 | | 1.2 ±1.0 | 1.1 ±0.8 | 1.1 ±0.9 | 1.1 ±0.9 |
| Other dairy | 0.3 ±0.5 | | 0.3 ±0.5 | 0.3 ±0.5 | 0.3 ±0.5 | 0.3 ±0.5 |
| Red meat | 0.8 ±0.7 | | 0.8 ±0.7 | 0.8 ±0.6 | 0.8 ±0.7 | 0.9 ±0.7 |
| Processed meat | 0.7 ±0.6 | | 0.7 ±0.7 | 0.8 ±0.6 | 0.7 ±0.6 | 0.8 ±0.7 |
| Poultry | 0.3 ±0.2 | | 0.2 ±0.2 | 0.2 ±0.2 | 0.2 ±0.2 | 0.2 ±0.2 |
| Fish and fish products | 0.4 ±0.3 | | 0.3 ±0.3 | 0.3 ±0.2 | 0.3 ±0.2 | 0.3 ±0.2 |
| Cereal and cereal products | 2.2 ±1.4 | | 2.4 ±1.4 | 2.4 ±1.3 | 2.2 ±1.3 | 2.2 ±1.3 |
| Potatoes | 0.4 ±0.3 | | 0.4 ±0.4 | 0.4 ±0.3 | 0.4 ±0.4 | 0.4 ±0.4 |
| Eggs and egg dishes | 0.4 ±0.4 | | 0.4 ±0.4 | 0.4 ±0.4 | 0.4 ±0.4 | 0.3 ±0.4 |
| Soups and sauces | 0.3 ±0.3 | | 0.3 ±0.3 | 0.3 ±0.4 | 0.3 ±0.3 | 0.3 ±0.3 |
| Fats and oils | 2.0 ±2.0 | | 2.3 ±2.1 | 2.4 ±2.1 | 2.3 ±2.1 | 2.7 ±2.3 |
| Sugars preserves and snacks | 1.3 ±1.2 | | 1.3 ±1.1 | 1.3 ±1.0 | 1.2 ±1.0 | 1.1 ±1.0 |
| Vegetables | 0.2 ±0.1 | | 0.2 ±0.1 | 0.2 ±0.1 | 0.2 ±0.1 | 0.2 ±0.1 |
| Red meat (g) | 36.5 ±29.8 | | 40.6 ±31.6 | 41.3 ±30.4 | 40.5 ±31.1 | 42.6 ±30.5 |
| Processed meat (g) | 25.0 ±21.4 | | 28.4 ±26.7 | 29.0 ±22.9 | 26.4 ±21.4 | 27.2 ±21.8 |
| Chicken (g) | 29.2 ±24.2 | | 27.7 ±21.5 | 26.8 ±19.9 | 25.4 ±21.8 | 21.1 ±18.8 |
| Milk (g) | 256.9 ±161.0 | | 284.9 ±164.6 | 324.8 ±100.6 | 412.8 ±141.3 | 473.8 ±149.2 |
| Yogurt (g) | 46.3 ±61.1 | | 39.8 ±56.8 | 39.5 ±51.1 | 35.0 ±48.2 | 25.3 ±42.2 |
| Cheese (g) | 20.7 ±21.6 | | 19.6 ±17.8 | 19.2 ±15.7 | 17.4 ±15.1 | 16.6 ±14.8 |
| Other dairy (g) | 7.2 ±14.3 | | 8.5 ±16.6 | 9.1 ±15.6 | 8.8 ±16.1 | 8.9 ±16.8 |
| Fruit and vegetables (g) | 525.6 ±278.9 | | 483.7 ±251.4 | 475.0 ±232.6 | 442.8 ±224.8 | 389.6 ±205.8 |

^1^Data are in means ±SD for continuous variables or in column percentages (%) for categorical variables. En%, percentage of total energy intake; EPIC, European Prospective Investigation into Cancer and Nutrition; Q, quintile; SFA, saturated fatty acids. ^2^Reference categories not shown for succinctness: sex: male; educational level: no; physical activity status: inactive; smoking status: never; hypertension: no; hypercholesterolemia: no; diabetes mellitus: no; cancer: no; hormone replacement therapy: no. ^3^Total missing values n (%): 1 (0.02)

# **SUPPLEMENTARY TABLE 5** Baseline characteristics across tertiles of saturated fatty acids intake from yogurt (expressed as percentage of total energy intake): 1993-1997, EPIC-Norfolk Study (n=21,841)^1^

|  | Q1  (n=7982) | Q2  (n=6579) | Q3  (n=7280) |
| --- | --- | --- | --- |
| Range of SFA from yogurt, en% | 0.0-0.0 | 0.0-0.1 | 0.1-6.0 |
| Age (years) | 60.0 ±9.3 | 57.8 ±9.2 | 57.6 ±8.8 |
| Sex^2^, female | 3,497 (43.8) | 3,886 (59.1) | 4,934 (67.8) |
| Educational level^2^ |  |  |  |
| Ordinary-level | 779 (9.8) | 720 (10.9) | 800 (11.0) |
| Advanced-level | 3,107 (38.9) | 2,733 (41.5) | 2,960 (40.7) |
| Degree | 705 (8.8) | 851 (12.9) | 1,346 (18.5) |
| Physical activity^2^ |  |  |  |
| Moderately inactive | 2,112 (26.5) | 1,980 (30.1) | 2,253 (31.0) |
| Moderately active | 1,697 (21.3) | 1,573 (23.9) | 1,811 (24.9) |
| Active | 1,478 (18.5) | 1,268 (19.3) | 1,396 (19.2) |
| Smoking status^2^ |  |  |  |
| Current | 1,344 (16.8) | 635 (9.7) | 591 (8.1) |
| Former | 3,449 (43.2) | 2,612 (39.7) | 2,830 (38.9) |
| Hormone replacement therapy (females only)^2^ | 639 (8.0) | 810 (12.3) | 1,084 (14.9) |
| Waist circumference (cm) | 89.7 ±12.3 | 87.6 ±12.2 | 85.6 ±11.8 |
| BMI (kg/m^2^) | 26.2 ±3.8 | 26.4 ±4.0 | 26.1 ±3.8 |
| Hypertension^2^ | 3,987 (50.0) | 3,033 (46.1) | 3,089 (42.4) |
| Hypercholesterolemia^2^ | 496 (6.2) | 497 (7.6) | 507 (7.0) |
| Diabetes mellitus^2^ | 157 (2.0) | 133 (2.0) | 118 (1.6) |
| Cancer^2,3^ | 415 (5.2) | 354 (5.4) | 417 (5.7) |
| Total energy (kcal) | 2,058.7 ±604.9 | 2,112.1 ±575.6 | 1,967.3 ±551.6 |
| Alcohol (g) | 9.4 ±14.7 | 8.6 ±12.7 | 8.0 ±10.9 |
| Dietary cholesterol (mg) | 294.7 ±123.4 | 282.2 ±111.0 | 253.3 ±103.2 |
| Fiber (g) | 17.2 ±6.2 | 19.4 ±6.4 | 19.4 ±6.5 |
| Protein (en %) | 16.0 ±3.1 | 16.6 ±2.9 | 17.5 ±3.1 |
| Carbohydrate (en %) | 49.3 ±6.9 | 50.6 ±6.3 | 51.3 ±6.4 |
| Total fat (en %) | 34.7 ±5.8 | 33.2 ±5.5 | 31.7 ±5.9 |
| PUFA (en %) | 6.0 ±2.1 | 6.4 ±2.0 | 6.2 ±1.9 |
| MUFA (en %) | 11.9 ±2.4 | 11.4 ±2.2 | 10.7 ±2.3 |
| Trans fatty acids (en %) | 1.2 ±0.5 | 1.1 ±0.4 | 1.0 ±0.4 |
| Total SFA (en %) | 13.7 ±3.4 | 12.5 ±2.9 | 12.0 ±3.2 |
| SFA from food source (en %) |  |  |  |
| Milk | 2.0 ±1.8 | 1.6 ±1.5 | 1.5 ±1.4 |
| Yogurt | 0.0 ±0.0 | 0.0 ±0.0 | 0.4 ±0.4 |
| Cheese | 1.1 ±0.9 | 1.1 ±0.9 | 1.2 ±0.9 |
| Other dairy | 0.3 ±0.6 | 0.3 ±0.5 | 0.3 ±0.5 |
| Red meat | 0.9 ±0.7 | 0.8 ±0.6 | 0.7 ±0.6 |
| Processed meat | 0.9 ±0.8 | 0.7 ±0.6 | 0.6 ±0.5 |
| Poultry | 0.2 ±0.2 | 0.2 ±0.2 | 0.2 ±0.2 |
| Fish and fish products | 0.3 ±0.3 | 0.3 ±0.2 | 0.3 ±0.3 |
| Cereal and cereal products | 2.4 ±1.5 | 2.4 ±1.3 | 2.0 ±1.2 |
| Potatoes | 0.5 ±0.4 | 0.4 ±0.3 | 0.3 ±0.3 |
| Eggs and egg dishes | 0.3 ±0.4 | 0.4 ±0.4 | 0.4 ±0.4 |
| Soups and sauces | 0.3 ±0.4 | 0.3 ±0.3 | 0.3 ±0.3 |
| Fats and oils | 2.7 ±2.4 | 2.2 ±1.9 | 2.1 ±1.9 |
| Sugars preserves and snacks | 1.3 ±1.2 | 1.3 ±1.0 | 1.1 ±1.0 |
| Vegetables | 0.2 ±0.1 | 0.2 ±0.1 | 0.2 ±0.1 |
| Red meat (g) | 45.3 ±32.9 | 41.0 ±29.8 | 34.2 ±28.0 |
| Processed meat (g) | 31.5 ±26.1 | 27.8 ±21.7 | 22.1 ±19.0 |
| Poultry (g) | 23.9 ±21.3 | 28.0 ±22.3 | 26.7 ±20.7 |
| Milk (g) | 350.7 ±175.4 | 359.9 ±160.6 | 342.3 ±160.3 |
| Yogurt (g) | 0.0 ±0.0 | 28.6 ±21.4 | 85.7 ±63.2 |
| Cheese (g) | 15.9 ±15.2 | 18.9 ±16.4 | 21.6 ±19.5 |
| Other dairy (g) | 8.0 ±17.1 | 9.2 ±15.8 | 8.4 ±14.6 |
| Fruit and vegetables (g) | 399.6 ±219.8 | 478.6 ±237.1 | 519.4 ±259.6 |

^1^Data are in means ±SD for continuous variables or in column percentages (%) for categorical variables. En%, percentage of total energy intake; EPIC, European Prospective Investigation into Cancer and Nutrition; Q, quintile; SFA, saturated fatty acids. ^2^Reference categories not shown for succinctness: sex: male; educational level: no; physical activity status: inactive; smoking status: never; hypertension: no; hypercholesterolemia: no; diabetes mellitus: no; cancer: no; hormone replacement therapy: no. ^3^Total missing values n (%): 1 (0.02)

# **SUPPLEMENTARY TABLE 6** Baseline characteristics across quintiles of saturated fatty acids intake from cheese (expressed as percentage of total energy intake): 1993-1997, EPIC-Norfolk Study (n=21,841)^1^

|  | Q1  (n=4369) | | Q2  (n=4368) | Q3  (n=4368) | Q4  (n=4368) | Q5  (n=4368) |
| --- | --- | --- | --- | --- | --- | --- |
| Range of SFA from cheese, en% | 0-0.4 | | 0.4-0.8 | 0.8-1.2 | 1.2-1.7 | 1.7-13.3 |
| Age (years) | 58.6 ±9.2 | | 58.4 ±9.0 | 58.8 ±9.3 | 58.3 ±9.2 | 58.5 ±9.2 |
| Sex^2^, female | 2,401 (55.0) | | 2,640 (60.4) | 2,104 (48.2) | 2,554 (58.5) | 2,554 (58.5) |
| Educational level^2^ |  | |  |  |  |  |
| Ordinary-level | 456 (10.4) | | 451 (10.3) | 471 (10.8) | 472 (10.8) | 472 (10.8) |
| Advanced-level | 1,718 (39.3) | | 1,707 (39.1) | 1,817 (41.6) | 1,798 (41.2) | 1,798 (41.2) |
| Degree | 446 (10.2) | | 466 (10.7) | 548 (12.6) | 630 (14.4) | 630 (14.4) |
| Physical activity^2^ |  | |  |  |  |  |
| Moderately inactive | | 1,262 (28.9) | 1,251 (28.6) | 1,226 (28.1) | 1,271 (29.1) | 1,271 (29.1) |
| Moderately active | 983 (22.5) | | 1,002 (22.9) | 1,042 (23.9) | 1,025 (23.5) | 1,025 (23.5) |
| Active | 758 (17.4) | | 865 (19.8) | 877 (20.1) | 849 (19.4) | 849 (19.4) |
| Smoking status^2^ |  | |  |  |  |  |
| Current | 575 (13.2) | | 559 (12.8) | 499 (11.4) | 452 (10.4) | 452 (10.4) |
| Former | 1,740 (39.8) | | 1,768 (40.5) | 1,823 (41.7) | 1,784 (40.8) | 1,784 (40.8) |
| Hormone replacement therapy (females only)^2^ | 503 (11.5) | | 541 (12.4) | 405 (9.3) | 531 (12.2) | 531 (12.2) |
| Waist circumference (cm) | 87.9 ±11.9 | | 87.4 ±12.2 | 88.6 ±12.0 | 87.7 ±12.4 | 86.8 ±12.5 |
| BMI (kg/m^2^) | 26.2 ±3.9 | | 26.4 ±3.9 | 26.1 ±3.7 | 26.4 ±3.9 | 26.0 ±3.9 |
| Hypertension^2^ | 2,105 (48.2) | | 2,032 (46.5) | 2,062 (47.2) | 1,994 (45.7) | 1,916 (43.9) |
| Hypercholesterolemia^2^ | 418 (9.6) | | 369 (8.5) | 257 (5.9) | 245 (5.6) | 211 (4.8) |
| Diabetes mellitus^2^ | 90 (2.1) | | 74 (1.7) | 59 (1.4) | 96 (2.2) | 89 (2.0) |
| Cancer^2,3^ | 252 (5.8) | | 241 (5.5) | 227 (5.2) | 228 (5.2) | 238 (5.5) |
| Total energy (kcal) | 2,037.3 ±599.5 | | 2,005.6 ±729.4 | 2,300.0 ±371.1 | 1,929.2 ±519.3 | 1,949.5 ±551.4 |
| Alcohol (g) | 7.9 ±13.3 | | 7.9 ±12.2 | 10.0 ±14.3 | 8.6 ±12.2 | 9.1 ±12.3 |
| Dietary cholesterol (mg) | 270.0 ±121.8 | | 272.6 ±130.8 | 317.0 ±97.2 | 263.3 ±104.4 | 262.8 ±106.5 |
| Fiber (g) | 18.4 ±6.7 | | 18.5 ±6.9 | 19.8 ±5.8 | 18.0 ±6.0 | 18.2 ±6.4 |
| Protein (en %) | 16.4 ±3.4 | | 16.9 ±3.4 | 15.9 ±2.7 | 17.1 ±2.9 | 17.0 ±3.0 |
| Carbohydrate (en %) | 52.0 ±7.1 | | 51.1 ±6.7 | 50.0 ±6.2 | 49.8 ±6.3 | 48.8 ±6.4 |
| Total fat (en %) | 32.2 ±6.3 | | 32.4 ±6.2 | 34.2 ±5.4 | 33.2 ±5.5 | 34.1 ±5.7 |
| PUFA (en %) | 6.2 ±2.1 | | 6.2 ±2.0 | 6.3 ±2.0 | 6.2 ±1.9 | 6.2 ±2.0 |
| MUFA (en %) | 11.2 ±2.6 | | 11.2 ±2.5 | 11.8 ±2.2 | 11.3 ±2.2 | 11.4 ±2.2 |
| Trans fatty acids (en %) | 1.1 ±0.5 | | 1.1 ±0.5 | 1.1 ±0.4 | 1.1 ±0.4 | 1.1 ±0.4 |
| Total SFA (en %) | 12.0 ±3.5 | | 12.2 ±3.3 | 13.2 ±3.1 | 12.8 ±3.1 | 13.6 ±3.1 |
| SFA from food source  (en %) |  | |  |  |  |  |
| Milk | 1.8 ±1.7 | | 1.8 ±1.7 | 1.7 ±1.5 | 1.7 ±1.6 | 1.7 ±1.6 |
| Yogurt | 0.1 ±0.3 | | 0.1 ±0.3 | 0.1 ±0.3 | 0.1 ±0.3 | 0.2 ±0.3 |
| Cheese | 0.2 ±0.1 | | 0.6 ±0.1 | 1.0 ±0.1 | 1.4 ±0.1 | 2.5 ±1.0 |
| Other dairy | 0.3 ±0.6 | | 0.3 ±0.5 | 0.3 ±0.5 | 0.3 ±0.5 | 0.3 ±0.5 |
| Red meat | 0.8 ±0.7 | | 0.9 ±0.7 | 0.8 ±0.6 | 0.8 ±0.7 | 0.8 ±0.6 |
| Processed meat | 0.8 ±0.7 | | 0.8 ±0.6 | 0.8 ±0.6 | 0.7 ±0.6 | 0.7 ±0.6 |
| Poultry | 0.3 ±0.2 | | 0.3 ±0.2 | 0.2 ±0.2 | 0.2 ±0.2 | 0.2 ±0.2 |
| Fish and fish products | 0.4 ±0.3 | | 0.4 ±0.3 | 0.3 ±0.2 | 0.3 ±0.2 | 0.3 ±0.2 |
| Cereal and cereal products | 2.4 ±1.5 | | 2.3 ±1.4 | 2.6 ±1.3 | 2.1 ±1.2 | 2.1 ±1.2 |
| Potatoes | 0.4 ±0.4 | | 0.4 ±0.4 | 0.4 ±0.3 | 0.4 ±0.3 | 0.3 ±0.3 |
| Eggs and egg dishes | 0.3 ±0.4 | | 0.4 ±0.4 | 0.4 ±0.3 | 0.4 ±0.4 | 0.4 ±0.4 |
| Soups and sauces | 0.3 ±0.4 | | 0.3 ±0.3 | 0.3 ±0.4 | 0.3 ±0.3 | 0.3 ±0.3 |
| Fats and oils | 2.3 ±2.2 | | 2.2 ±2.1 | 2.6 ±2.3 | 2.3 ±2.1 | 2.3 ±2.0 |
| Sugars preserves and snacks | 1.3 ±1.2 | | 1.2 ±1.1 | 1.3 ±1.0 | 1.2 ±1.0 | 1.2 ±1.0 |
| Vegetables | 0.2 ±0.1 | | 0.2 ±0.1 | 0.2 ±0.1 | 0.2 ±0.1 | 0.2 ±0.1 |
| Red meat (g) | 41.9 ±33.2 | | 41.3 ±32.8 | 45.5 ±30.3 | 38.4 ±28.4 | 34.5 ±27.5 |
| Processed meat (g) | 28.2 ±24.8 | | 27.7 ±24.6 | 31.9 ±22.2 | 25.7 ±21.1 | 22.7 ±20.7 |
| Poultry (g) | 28.1 ±22.8 | | 26.9 ±22.4 | 27.1 ±19.2 | 25.5 ±22.1 | 22.7 ±20.4 |
| Milk (g) | 356.5 ±173.8 | | 350.6 ±164.3 | 372.5 ±162.1 | 345.4 ±157.9 | 328.2 ±169.4 |
| Yogurt (g) | 33.6 ±55.6 | | 36.8 ±50.8 | 36.9 ±50.6 | 37.9 ±50.9 | 40.8 ±55.3 |
| Cheese (g) | 4.0 ±5.0 | | 12.5 ±10.8 | 18.4 ±8.7 | 21.6 ±11.1 | 36.9 ±23.6 |
| Other dairy (g) | 9.2 ±17.9 | | 8.9 ±16.8 | 9.6 ±15.8 | 7.5 ±13.9 | 7.3 ±14.7 |
| Fruit and vegetables (g) | 452.9 ±261.6 | | 469.8 ±251.3 | 468.7 ±231.7 | 459.1 ±231.6 | 466.1 ±243.4 |

^1^Data are in means ±SD for continuous variables or in column percentages (%) for categorical variables. En%, percentage of total energy intake; EPIC, European Prospective Investigation into Cancer and Nutrition; Q, quintile; SFA, saturated fatty acids. ^2^Reference categories not shown for succinctness: sex: male; educational level: no; physical activity status: inactive; smoking status: never; hypertension: no; hypercholesterolemia: no; diabetes mellitus: no; cancer: no; hormone replacement therapy: no. ^3^Total missing values n (%): 1 (0.02)

# **SUPPLEMENTARY TABLE 7** Baseline characteristics across quintiles of saturated fatty acids intake from red meat (expressed as percentage of total energy intake): 1993-1997, EPIC-Norfolk Study (n=21,841)^1^

|  | | Q1  (n=4369) | Q2  (n=4368) | Q3  (n=4368) | Q4  (n=4368) | Q5  (n=4368) |
| --- | --- | --- | --- | --- | --- | --- |
| Range SFA from red meat, en% | | 0-0.3 | 0.3-0.5 | 0.5-0.8 | 0.8-1.2 | 1.2-9.8 |
| Age (years) | | 58.5 ±9.1 | 59.3 ±9.2 | 58.6 ±9.1 | 58.3 ±9.3 | 57.9 ±9.2 |
| Sex^2^, female | | 2,635 (60.3) | 2,587 (59.2) | 2,489 (57.0) | 2,431 (55.7) | 2,175 (49.8) |
| Educational level^2^ | |  |  |  |  |  |
| Ordinary-level | | 463 (10.6) | 419 (9.6) | 465 (10.7) | 480 (11.0) | 472 (10.8) |
| Advanced-level | | 1,741 (39.9) | 1,740 (39.8) | 1,731 (39.6) | 1,784 (40.8) | 1,804 (41.3) |
| Degree | | 702 (16.1) | 608 (13.9) | 555 (12.7) | 526 (12.0) | 511 (11.7) |
| Physical activity^2^ | |  |  |  |  |  |
| Moderately inactive | 1,284 (29.4) | | 1,315 (30.1) | 1,283 (29.4) | 1,246 (28.5) | 1,217 (27.9) |
| Moderately active | | 1,011 (23.1) | 1,047 (24.0) | 977 (22.4) | 1,043 (23.9) | 1,003 (23.0) |
| Active | | 845 (19.3) | 790 (18.1) | 817 (18.7) | 839 (19.2) | 851 (19.5) |
| Smoking status^2^ | |  |  |  |  |  |
| Current | | 392 (9.0) | 437 (10.0) | 465 (10.7) | 539 (12.3) | 737 (16.9) |
| Former | | 1,728 (39.6) | 1,774 (40.6) | 1,787 (40.9) | 1,785 (40.9) | 1,817 (41.6) |
| Hormone replacement therapy (females only)^2^ | | 517 (11.8) | 523 (12.0) | 512 (11.7) | 488 (11.2) | 493 (11.3) |
| Waist circumference (cm) | | 85.8 ±11.9 | 87.0 ±11.8 | 87.7 ±12.2 | 88.2 ±12.2 | 89.8 ±12.6 |
| BMI (kg/m^2^) | | 25.6 ±3.8 | 26.1 ±3.9 | 26.3 ±3.9 | 26.4 ±3.8 | 26.7 ±3.9 |
| Hypertension^2^ | | 1,987 (45.5) | 2,060 (47.2) | 2,075 (47.5) | 2,026 (46.4) | 1,961 (44.9) |
| Hypercholesterolemia^2^ | | 368 (8.4) | 319 (7.3) | 308 (7.1) | 263 (6.0) | 242 (5.5) |
| Diabetes mellitus^2^ | | 89 (2.0) | 69 (1.6) | 75 (1.7) | 89 (2.0) | 86 (2.0) |
| Cancer^2,3^ | | 268 (6.1) | 224 (5.1) | 225 (5.2) | 239 (5.5) | 230 (5.3) |
| Total energy (kcal) | | 2,041.9 ±584.0 | 2,094.5 ±603.8 | 2,063.8 ±579.2 | 2,023.8 ±567.4 | 1,997.7 ±568.8 |
| Alcohol (g) | | 7.5 ±11.9 | 8.4 ±12.7 | 8.5 ±12.8 | 9.1 ±12.9 | 10.0 ±14.1 |
| Dietary cholesterol (mg) | | 232.6 ±106.9 | 267.1 ±109.8 | 279.1 ±108.4 | 292.7 ±113.5 | 314.1 ±117.8 |
| Fiber (g) | | 20.5 ±7.2 | 19.4 ±6.3 | 18.6 ±6.2 | 17.8 ±5.7 | 16.5 ±5.8 |
| Protein (en %) | | 15.5 ±3.1 | 15.9 ±2.9 | 16.6 ±2.9 | 17.2 ±3.0 | 18.1 ±3.1 |
| Carbohydrate (en %) | | 53.7 ±6.5 | 52.2 ±6.1 | 50.8 ±5.8 | 49.1 ±5.8 | 46.0 ±6.1 |
| Total fat (en %) | | 31.6 ±6.4 | 32.4 ±5.9 | 33.0 ±5.5 | 33.7 ±5.5 | 35.4 ±5.5 |
| PUFA (en %) | | 6.5 ±2.3 | 6.2 ±2.1 | 6.2 ±2.0 | 6.1 ±1.9 | 6.0 ±1.8 |
| MUFA (en %) | | 10.6 ±2.5 | 11.0 ±2.4 | 11.2 ±2.2 | 11.6 ±2.2 | 12.4 ±2.2 |
| Trans fatty acids (en %) | | 1.0 ±0.5 | 1.1 ±0.5 | 1.1 ±0.4 | 1.1 ±0.4 | 1.1 ±0.4 |
| Total SFA (en %) | | 11.7 ±3.4 | 12.3 ±3.1 | 12.7 ±3.1 | 13.1 ±3.1 | 14.0 ±3.1 |
| SFA from food source  (en %) | |  |  |  |  |  |
| Milk | | 1.6 ±1.6 | 1.7 ±1.5 | 1.7 ±1.6 | 1.8 ±1.6 | 1.9 ±1.7 |
| Yogurt | | 0.2 ±0.4 | 0.1 ±0.3 | 0.1 ±0.3 | 0.1 ±0.3 | 0.1 ±0.3 |
| Cheese | | 1.2 ±1.1 | 1.1 ±0.9 | 1.2 ±0.9 | 1.1 ±0.8 | 1.1 ±0.8 |
| Other dairy | | 0.3 ±0.5 | 0.3 ±0.5 | 0.3 ±0.5 | 0.3 ±0.5 | 0.3 ±0.5 |
| Red meat | | 0.2 ±0.1 | 0.4 ±0.1 | 0.7 ±0.1 | 1.0 ±0.1 | 1.9 ±0.7 |
| Processed meat | | 0.4 ±0.5 | 0.6 ±0.5 | 0.7 ±0.6 | 0.9 ±0.6 | 1.1 ±0.8 |
| Poultry | | 0.2 ±0.2 | 0.2 ±0.2 | 0.2 ±0.2 | 0.2 ±0.2 | 0.2 ±0.2 |
| Fish and fish products | | 0.4 ±0.3 | 0.3 ±0.3 | 0.3 ±0.2 | 0.3 ±0.2 | 0.3 ±0.2 |
| Cereal and cereal products | | 2.3 ±1.4 | 2.4 ±1.4 | 2.3 ±1.3 | 2.3 ±1.3 | 2.0 ±1.2 |
| Potatoes | | 0.3 ±0.4 | 0.4 ±0.3 | 0.4 ±0.4 | 0.4 ±0.4 | 0.5 ±0.4 |
| Eggs and egg dishes | | 0.4 ±0.4 | 0.3 ±0.4 | 0.3 ±0.3 | 0.4 ±0.4 | 0.4 ±0.4 |
| Soups and sauces | | 0.2 ±0.3 | 0.3 ±0.3 | 0.3 ±0.3 | 0.3 ±0.3 | 0.3 ±0.4 |
| Fats and oils | | 2.3 ±2.2 | 2.4 ±2.1 | 2.4 ±2.1 | 2.3 ±2.0 | 2.4 ±2.2 |
| Sugars preserves and snacks | | 1.2 ±1.1 | 1.3 ±1.1 | 1.3 ±1.1 | 1.2 ±1.0 | 1.2 ±1.0 |
| Vegetables | | 0.2 ±0.2 | 0.2 ±0.1 | 0.2 ±0.1 | 0.2 ±0.1 | 0.2 ±0.1 |
| Red meat (g) | | 9.9 ±8.9 | 26.4 ±13.0 | 38.5 ±16.8 | 51.1 ±20.3 | 75.6 ±36.1 |
| Processed meat (g) | | 18.3 ±21.6 | 25.0 ±20.1 | 28.2 ±21.0 | 30.2 ±21.4 | 34.5 ±26.7 |
| Poultry (g) | | 25.6 ±27.8 | 26.6 ±19.7 | 26.5 ±19.1 | 26.2 ±20.5 | 25.3 ±19.0 |
| Milk (g) | | 355.9 ±171.4 | 361.4 ±161.7 | 353.8 ±162.4 | 345.7 ±165.3 | 336.5 ±169.0 |
| Yogurt (g) | | 47.8 ±65.2 | 41.2 ±54.8 | 37.9 ±50.8 | 32.9 ±46.2 | 26.1 ±40.8 |
| Cheese (g) | | 21.7 ±21.9 | 19.2 ±17.8 | 18.9 ±16.7 | 17.6 ±14.3 | 16.1 ±13.8 |
| Other dairy (g) | | 8.5 ±17.5 | 9.5 ±17.9 | 8.7 ±15.4 | 8.3 ±14.5 | 7.6 ±13.6 |
| Fruit and vegetables (g) | | 513.2 ±279.0 | 484.6 ±249.5 | 460.5 ±235.6 | 442.8 ±221.2 | 415.5 ±219.3 |

^1^Data are in means ±SD for continuous variables or in column percentages (%) for categorical variables. En%, percentage of total energy intake; EPIC, European Prospective Investigation into Cancer and Nutrition; Q, quintile; SFA, saturated fatty acids. ^2^Reference categories not shown for succinctness: sex: male; educational level: no; physical activity status: inactive; smoking status: never; hypertension: no; hypercholesterolemia: no; diabetes mellitus: no; cancer: no; hormone replacement therapy: no. ^3^Total missing values n (%): 1 (0.02).

# **SUPPLEMENTARY TABLE 8** Baseline characteristics across quintiles of saturated fatty acids intake from processed meat (expressed as percentage of total energy intake): 1993-1997, EPIC-Norfolk Study (n=21,841)^1^

|  | | Q1  (n=4369) | Q2  (n=4368) | Q3  (n=4368) | Q4  (n=4368) | Q5  (n=4368) |
| --- | --- | --- | --- | --- | --- | --- |
| Range SFA from processed meat, en% | | 0.0-0.2 | 0.2-0.5 | 0.5-0.7 | 0.7-1.1 | 1.1-10.6 |
| Age (years) | | 58.1 ±9.1 | 59.2 ±9.3 | 58.7 ±9.1 | 58.4 ±9.2 | 58.3 ±9.2 |
| Sex^2^, female | | 3,023 (69.2) | 2,678 (61.3) | 2,563 (58.7) | 2,251 (51.5) | 1,802 (41.3) |
| Educational level^2^ | |  |  |  |  |  |
| Ordinary-level | | 445 (10.2) | 469 (10.7) | 462 (10.6) | 478 (10.9) | 445 (10.2) |
| Advanced-level | | 1,779 (40.7) | 1,815 (41.6) | 1,763 (40.4) | 1,723 (39.5) | 1,720 (39.4) |
| Degree | | 848 (19.4) | 650 (14.9) | 562 (12.9) | 436 (10.0) | 406 (9.3) |
| Physical activity^2^ | |  |  |  |  |  |
| Moderately inactive | 1,315 (30.1) | | 1,334 (30.5) | 1,352 (31.0) | 1,198 (27.4) | 1,146 (26.2) |
| Moderately active | | 1,059 (24.2) | 1,046 (24.0) | 1,015 (23.2) | 994 (22.8) | 967 (22.1) |
| Active | | 885 (20.3) | 757 (17.3) | 794 (18.2) | 839 (19.2) | 867 (19.9) |
| Smoking status^2^ | |  |  |  |  |  |
| Current | | 389 (8.9) | 420 (9.6) | 495 (11.3) | 520 (11.9) | 746 (17.1) |
| Former | | 1,702 (39.0) | 1,745 (40.0) | 1,719 (39.4) | 1,834 (42.0) | 1,891 (43.3) |
| Hormone replacement therapy (females only)^2^ | | 670 (15.3) | 566 (13.0) | 539 (12.3) | 425 (9.7) | 333 (7.6) |
| Waist circumference (cm) | | 84.1 ±11.7 | 86.4 ±11.6 | 87.3 ±12.0 | 89.2 ±12.2 | 91.5 ±12.3 |
| BMI (kg/m^2^) | | 25.5 ±3.8 | 26.0 ±3.6 | 26.3 ±3.8 | 26.6 ±3.9 | 26.9 ±4.0 |
| Hypertension^2^ | | 1,874 (42.9) | 1,985 (45.4) | 2,053 (47.0) | 2,104 (48.2) | 2,093 (47.9) |
| Hypercholesterolemia^2^ | | 399 (9.1) | 293 (6.7) | 306 (7.0) | 274 (6.3) | 228 (5.2) |
| Diabetes mellitus^2^ | | 78 (1.8) | 76 (1.7) | 62 (1.4) | 85 (2.0) | 107 (2.5) |
| Cancer^2,3^ | | 272 (6.2) | 256 (5.9) | 216 (5.0) | 227 (5.2) | 215 (4.9) |
| Total energy (kcal) | | 1,946.5±567.2 | 2,069.2 ±582.9 | 2,077.0 ±569.9 | 2,060.6 ±557.7 | 2,068.5 ±618.6 |
| Alcohol (g) | | 7.9 ±11.5 | 9.1 ±13.3 | 8.6 ±12.6 | 8.5 ±12.8 | 9.4 ±14.2 |
| Dietary cholesterol (mg) | | 226.6 ±104.0 | 267.4 ±108.3 | 281.1 ±106.0 | 292.8 ±106.0 | 317.7 ±126.9 |
| Fiber (g) | | 20.5 ±7.2 | 19.5 ±6.4 | 18.6 ±6.0 | 17.8 ±5.8 | 16.5 ±5.7 |
| Protein (en %) | | 16.6 ±3.4 | 16.5 ±3.1 | 16.5 ±3.1 | 16.7 ±3.0 | 17.0 ±3.0 |
| Carbohydrate (en %) | | 53.3 ±6.8 | 51.6 ±6.3 | 50.6 ±6.0 | 49.5 ±5.9 | 46.8 ±6.3 |
| Total fat (en %) | | 30.7 ±6.3 | 32.1 ±5.8 | 33.2 ±5.4 | 34.1 ±5.2 | 36.1 ±5.3 |
| PUFA (en %) | | 6.4 ±2.2 | 6.2 ±2.1 | 6.2 ±2.0 | 6.1 ±1.9 | 6.2 ±1.8 |
| MUFA (en %) | | 10.2 ±2.4 | 10.8 ±2.2 | 11.3 ±2.1 | 11.8 ±2.1 | 12.7 ±2.1 |
| Trans fatty acids (en %) | | 0.9 ±0.5 | 1.0 ±0.5 | 1.1 ±0.4 | 1.2 ±0.4 | 1.2 ±0.4 |
| Total SFA (en %) | | 11.4 ±3.4 | 12.3 ±3.3 | 12.8 ±3.1 | 13.2 ±3.0 | 14.1 ±3.0 |
| SFA from food source  (en %) | |  |  |  |  |  |
| Milk | | 1.6 ±1.7 | 1.7 ±1.6 | 1.7 ±1.6 | 1.8 ±1.5 | 1.8 ±1.7 |
| Yogurt | | 0.2 ±0.4 | 0.2 ±0.3 | 0.1 ±0.3 | 0.1 ±0.3 | 0.1 ±0.2 |
| Cheese | | 1.3 ±1.1 | 1.1 ±0.9 | 1.1 ±0.8 | 1.1 ±0.9 | 1.1 ±0.8 |
| Other dairy | | 0.3 ±0.5 | 0.3 ±0.6 | 0.3 ±0.5 | 0.3 ±0.5 | 0.3 ±0.5 |
| Red meat | | 0.5 ±0.5 | 0.7 ±0.5 | 0.8 ±0.6 | 1.0 ±0.7 | 1.2 ±0.8 |
| Processed meat | | 0.1 ±0.1 | 0.4 ±0.1 | 0.6 ±0.1 | 0.9 ±0.1 | 1.8 ±0.7 |
| Poultry | | 0.2 ±0.2 | 0.2 ±0.2 | 0.2 ±0.2 | 0.2 ±0.2 | 0.2 ±0.2 |
| Fish and fish products | | 0.3 ±0.3 | 0.3 ±0.2 | 0.3 ±0.2 | 0.3 ±0.2 | 0.4 ±0.2 |
| Cereal and cereal products | | 2.1 ±1.3 | 2.3 ±1.4 | 2.4 ±1.4 | 2.4 ±1.3 | 2.2 ±1.3 |
| Potatoes | | 0.3 ±0.3 | 0.3 ±0.3 | 0.4 ±0.4 | 0.4 ±0.4 | 0.5 ±0.4 |
| Eggs and egg dishes | | 0.4 ±0.4 | 0.3 ±0.4 | 0.3 ±0.4 | 0.4 ±0.4 | 0.4 ±0.4 |
| Soups and sauces | | 0.3 ±0.3 | 0.3 ±0.3 | 0.3 ±0.4 | 0.3 ±0.3 | 0.3 ±0.3 |
| Fats and oils | | 2.3 ±2.1 | 2.4 ±2.2 | 2.4 ±2.1 | 2.3 ±2.1 | 2.3 ±2.1 |
| Sugars preserves and snacks | | 1.1 ±1.1 | 1.2 ±1.1 | 1.3 ±1.1 | 1.3 ±1.0 | 1.3 ±1.1 |
| Vegetables | | 0.2 ±0.2 | 0.2 ±0.1 | 0.2 ±0.1 | 0.2 ±0.1 | 0.2 ±0.1 |
| Red meat (g) | | 23.2 ±26.6 | 36.9 ±27.3 | 42.2 ±30.2 | 46.8 ±28.1 | 52.4 ±32.9 |
| Processed meat (g) | | 5.6 ±5.6 | 16.4 ±7.6 | 24.4 ±9.4 | 33.3 ±11.5 | 56.5 ±28.9 |
| Poultry (g) | | 26.1 ±28.3 | 28.2 ±20.5 | 26.8 ±19.3 | 25.1 ±18.9 | 23.9 ±18.6 |
| Milk (g) | | 348.7 ±170.6 | 362.8 ±166.4 | 350.8 ±163.1 | 354.2 ±162.4 | 336.9 ±167.5 |
| Yogurt (g) | | 52.7 ±67.9 | 41.5 ±51.9 | 36.9 ±50.2 | 30.3 ±43.0 | 24.5 ±42.0 |
| Cheese (g) | | 22.4 ±22.8 | 19.2 ±17.6 | 18.3 ±14.8 | 17.4 ±14.4 | 16.2 ±14.5 |
| Other dairy (g) | | 7.8 ±15.9 | 8.7 ±16.0 | 8.9 ±16.3 | 8.7 ±16.0 | 8.3 ±15.3 |
| Fruit and vegetables (g) | | 544.7 ±287.2 | 494.3 ±249.5 | 458.0 ±226.0 | 429.5 ±212.8 | 390.2 ±207.7 |

^1^Data are in means ±SD for continuous variables or in column percentages (%) for categorical variables. En%, percentage of total energy intake; EPIC, European Prospective Investigation into Cancer and Nutrition; Q, quintile; SFA, saturated fatty acids. ^2^Reference categories not shown for succinctness: sex: male; educational level: no; physical activity status: inactive; smoking status: never; hypertension: no; hypercholesterolemia: no; diabetes mellitus: no; cancer: no; hormone replacement therapy: no. ^3^Total missing values n (%): 1 (0.02).

# **SUPPLEMENTARY TABLE 9** Baseline characteristics across quintiles of saturated fatty acids intake from poultry (expressed as percentage of total energy intake): 1993-1997, EPIC-Norfolk Study (n=21,841)^1^

|  | Q1  (n=4369) | | Q2  (n=4368) | Q3  (n=4368) | Q4  (n=4368) | Q5  (n=4368) |
| --- | --- | --- | --- | --- | --- | --- |
| Range SFA from poultry, en% | 0.0-0.1 | | 0.1-0.1 | 0.1-0.2 | 0.2-0.4 | 0.4-3.1 |
| Age (years) | 59.7 ±9.5 | | 59.1 ±9.3 | 58.6 ±9.1 | 57.8 ±9.1 | 57.5 ±8.8 |
| Sex^2^, female | 2,123 (48.6) | | 2,158 (49.4) | 2,680 (61.4) | 2,348 (53.8) | 3,008 (68.9) |
| Educational level^2^ |  | |  |  |  |  |
| Ordinary-level | 411 (9.4) | | 439 (10.1) | 467 (10.7) | 466 (10.7) | 516 (11.8) |
| Advanced-level | 1,729 (39.6) | | 1,724 (39.5) | 1,766 (40.4) | 1,795 (41.1) | 1,786 (40.9) |
| Degree | 647 (14.8) | | 563 (12.9) | 551 (12.6) | 586 (13.4) | 555 (12.7) |
| Physical activity^2^ |  | |  |  |  |  |
| Moderately inactive | | 1,216 (27.8) | 1,248 (28.6) | 1,249 (28.6) | 1,270 (29.1) | 1,362 (31.2) |
| Moderately active | 998 (22.8) | | 969 (22.2) | 1,015 (23.2) | 1,058 (24.2) | 1,041 (23.8) |
| Active | 875 (20.0) | | 849 (19.4) | 813 (18.6) | 865 (19.8) | 740 (16.9) |
| Smoking status^2^ |  | |  |  |  |  |
| Current | 541 (12.4) | | 611 (14.0) | 506 (11.6) | 475 (10.9) | 437 (10.0) |
| Former | 1,803 (41.3) | | 1,780 (40.8) | 1,744 (39.9) | 1,792 (41.0) | 1,772 (40.6) |
| Hormone replacement therapy (females only)^2^ | 374 (8.6) | | 399 (9.1) | 567 (13.0) | 479 (11.0) | 714 (16.4) |
| Waist circumference (cm) | 88.5 ±12.3 | | 88.7 ±12.0 | 87.1 ±12.3 | 87.9 ±12.0 | 86.3 ±12.4 |
| BMI (kg/m^2^) | 26.0 ±3.8 | | 26.2 ±3.7 | 26.3 ±3.8 | 26.1 ±3.8 | 26.5 ±4.1 |
| Hypertension^2^ | 2,087 (47.8) | | 2,090 (47.9) | 2,028 (46.4) | 1,944 (44.5) | 1,960 (44.9) |
| Hypercholesterolemia^2^ | 232 (5.3) | | 252 (5.8) | 294 (6.7) | 289 (6.6) | 433 (9.9) |
| Diabetes mellitus^2^ | 65 (1.5) | | 81 (1.9) | 102 (2.3) | 59 (1.4) | 101 (2.3) |
| Cancer^2,3^ | 226 (5.2) | | 211 (4.8) | 220 (5.0) | 256 (5.9) | 273 (6.3) |
| Total energy (kcal) | 2,224.5 ±623.2 | | 2,214.2 ±576.4 | 1,753.6 ±291.7 | 2,310.6 ±679.6 | 1,718.7 ±318.8 |
| Alcohol (g) | 8.3 ±13.4 | | 9.5 ±14.4 | 8.0 ±11.4 | 9.7 ±13.7 | 7.9 ±11.2 |
| Dietary cholesterol (mg) | 283.9 ±130.3 | | 296.4 ±118.1 | 234.3 ±78.9 | 323.6 ±129.4 | 247.5 ±80.4 |
| Fiber (g) | 19.6 ±7.0 | | 18.9 ±6.5 | 16.8 ±5.1 | 20.1 ±7.1 | 17.4 ±5.5 |
| Protein (en %) | 14.7 ±2.6 | | 15.3 ±2.5 | 16.9 ±2.4 | 16.9 ±2.7 | 19.5 ±3.0 |
| Carbohydrate (en %) | 51.4 ±6.8 | | 50.5 ±6.7 | 50.5 ±6.5 | 49.7 ±6.4 | 49.7 ±6.6 |
| Total fat (en %) | 34.6 ±5.9 | | 34.5 ±5.8 | 32.7 ±5.7 | 33.6 ±5.7 | 30.8 ±5.7 |
| PUFA (en %) | 6.2 ±2.2 | | 6.2 ±2.1 | 6.1 ±1.9 | 6.4 ±2.0 | 6.2 ±1.9 |
| MUFA (en %) | 11.8 ±2.4 | | 11.8 ±2.4 | 11.1 ±2.2 | 11.6 ±2.3 | 10.5 ±2.2 |
| Trans fatty acids (en %) | 1.2 ±0.5 | | 1.2 ±0.5 | 1.1 ±0.4 | 1.1 ±0.4 | 0.9 ±0.4 |
| Total SFA (en %) | 13.6 ±3.5 | | 13.5 ±3.2 | 12.6 ±3.1 | 12.7 ±3.1 | 11.3 ±3.0 |
| SFA from food source  (en %) |  | |  |  |  |  |
| Milk | 1.9 ±1.7 | | 1.9 ±1.7 | 1.8 ±1.6 | 1.5 ±1.4 | 1.4 ±1.4 |
| Yogurt | 0.1 ±0.3 | | 0.1 ±0.3 | 0.1 ±0.3 | 0.1 ±0.3 | 0.2 ±0.3 |
| Cheese | 1.3 ±1.1 | | 1.1 ±0.9 | 1.2 ±0.9 | 1.0 ±0.8 | 1.1 ±0.9 |
| Other dairy | 0.3 ±0.5 | | 0.3 ±0.5 | 0.3 ±0.5 | 0.3 ±0.5 | 0.3 ±0.5 |
| Red meat | 0.7 ±0.7 | | 0.8 ±0.7 | 0.9 ±0.7 | 0.8 ±0.7 | 0.8 ±0.7 |
| Processed meat | 0.7 ±0.7 | | 0.8 ±0.7 | 0.8 ±0.6 | 0.8 ±0.7 | 0.6 ±0.6 |
| Poultry | 0.0 ±0.0 | | 0.1 ±0.0 | 0.2 ±0.0 | 0.3 ±0.1 | 0.5 ±0.2 |
| Fish and fish products | 0.3 ±0.3 | | 0.3 ±0.2 | 0.3 ±0.2 | 0.3 ±0.3 | 0.4 ±0.3 |
| Cereal and cereal products | 2.7 ±1.5 | | 2.6 ±1.4 | 2.1 ±1.2 | 2.3 ±1.3 | 1.8 ±1.1 |
| Potatoes | 0.4 ±0.4 | | 0.4 ±0.4 | 0.4 ±0.4 | 0.4 ±0.3 | 0.4 ±0.3 |
| Eggs and egg dishes | 0.4 ±0.4 | | 0.4 ±0.4 | 0.4 ±0.4 | 0.3 ±0.3 | 0.3 ±0.4 |
| Soups and sauces | 0.3 ±0.3 | | 0.3 ±0.4 | 0.3 ±0.3 | 0.3 ±0.4 | 0.3 ±0.3 |
| Fats and oils | 2.8 ±2.4 | | 2.6 ±2.2 | 2.2 ±2.0 | 2.4 ±2.1 | 1.8 ±1.7 |
| Sugars preserves and snacks | 1.3 ±1.2 | | 1.3 ±1.1 | 1.2 ±1.0 | 1.3 ±1.1 | 1.1 ±1.0 |
| Vegetables | 0.2 ±0.1 | | 0.2 ±0.1 | 0.2 ±0.1 | 0.2 ±0.1 | 0.2 ±0.1 |
| Red meat (g) | 36.4 ±35.2 | | 43.3 ±30.4 | 39.4 ±24.7 | 46.5 ±32.1 | 36.0 ±29.0 |
| Processed meat (g) | 26.6 ±27.0 | | 31.0 ±23.7 | 25.1 ±17.7 | 31.8 ±25.7 | 21.7 ±17.1 |
| Poultry (g) | 5.6 ±4.4 | | 13.7 ±3.7 | 16.0 ±2.4 | 42.3 ±13.7 | 52.5 ±21.3 |
| Milk (g) | 369.9 ±174.0 | | 369.5 ±166.6 | 335.7 ±156.2 | 362.3 ±168.9 | 315.9 ±158.0 |
| Yogurt (g) | 34.6 ±53.7 | | 34.2 ±50.5 | 35.0 ±49.3 | 39.5 ±54.4 | 42.7 ±55.0 |
| Cheese (g) | 21.6 ±21.1 | | 19.3 ±17.3 | 17.0 ±15.0 | 19.0 ±16.3 | 16.6 ±15.5 |
| Other dairy (g) | 9.3 ±17.3 | | 9.9 ±17.6 | 7.0 ±13.4 | 10.0 ±17.8 | 6.4 ±12.2 |
| Fruit and vegetables (g) | 454.6 ±254.6 | | 448.8 ±248.2 | 436.5 ±215.2 | 495.8 ±267.3 | 481.0 ±227.6 |

^1^Data are in means ±SD for continuous variables or in column percentages (%) for categorical variables. En%, percentage of total energy intake; EPIC, European Prospective Investigation into Cancer and Nutrition; Q, quintile; SFA, saturated fatty acids. ^2^Reference categories not shown for succinctness: sex: male; educational level: no; physical activity status: inactive; smoking status: never; hypertension: no; hypercholesterolemia: no; diabetes mellitus: no; cancer: no; hormone replacement therapy: no. ^3^Total missing values n (%): 1 (0.02).

# **SUPPLEMENTARY TABLE 10** Hazard ratios and 95% confidence intervals for risk of overall and subtypes of cardiovascular disease associated with the Modeled replacement of saturated fatty acids from total and types of meat by total and types of dairy in the EPIC-Norfolk Study (n=21,841)^1^

| Outcomes and  SFA from food to be replaced  (per 2.5 en %) | SFA from replacement food  (per 2.5 en %) | Unadjusted | Age, sex, energy-adjusted | Sociodemographic, lifestyle and energy-adjusted | Sociodemographic, lifestyle, energy and cardiometabolic characteristics-adjusted |
| --- | --- | --- | --- | --- | --- |
|  |  | HR (95% CI) | HR (95% CI) | HR (95% CI) | HR (95% CI) |
| CVD (5,902 cases/395,854.5 person-years) | | | | | |
| Total meat | Total dairy | 0.90 (0.84, 0.95) | 0.83 (0.78,0.89) | 0.88 (0.82,0.94) | 0.91 (0.85,0.98) |
| Red meat | Milk | 1.00 (0.91,1.11) | 0.89 (0.80,0.98) | 0.91 (0.82,1.00) | 0.94 (0.85,1.04) |
|  | Cheese | 0.77 (0.68,0.86) | 0.75 (0.67,0.85) | 0.84 (0.75,0.94) | 0.86 (0.77,0.97) |
|  | Yogurt | 0.46 (0.35,0.60) | 0.72 (0.57,0.92) | 0.91 (0.72,1.16) | 0.98 (0.78,1.24) |
| Processed meat | Milk | 0.78 (0.71,0.86) | 0.76 (0.69,0.84) | 0.81 (0.73,0.90) | 0.86 (0.78,0.96) |
|  | Cheese | 0.61 (0.55,0.68) | 0.65 (0.58,0.73) | 0.75 (0.66,0.84) | 0.79 (0.70,0.90) |
|  | Yogurt | 0.40 (0.31,0.52) | 0.65 (0.51,0.82) | 0.83 (0.66,1.05) | 0.92 (0.73,1.16) |
| Poultry | Milk | 3.66 (2.57,5.21) | 1.19 (0.84,1.69) | 1.07 (0.75,1.51) | 1.24 (0.88,1.76) |
|  | Cheese | 3.33 (2.33,4.76) | 1.08 (0.75,1.54) | 1.00 (0.70,1.42) | 1.17 (0.82,1.67) |
|  | Yogurt | 1.86 (1.19,2.88) | 0.95 (0.63,1.45) | 1.04 (0.69,1.57) | 1.29 (0.86,1.95) |
| CAD (4,215 cases/401,846.7 person-years) | | | | | |
| Total meat | Total dairy | 0.84 (0.78,0.90) | 0.82 (0.76,0.88) | 0.87 (0.80,0.94) | 0.92 (0.85,0.99) |
| Red meat | Milk | 0.95 (0.85,1.08) | 0.88 (0.78,0.99) | 0.90 (0.80,1.01) | 0.94 (0.84,1.07) |
|  | Cheese | 0.73 (0.64,0.84) | 0.75 (0.66,0.86) | 0.85 (0.74,0.98) | 0.89 (0.77,1.02) |
|  | Yogurt | 0.39 (0.28,0.54) | 0.68 (0.50,0.91) | 0.91 (0.68,1.21) | 1.00 (0.75,1.33) |
| Processed meat | Milk | 0.71 (0.64,0.79) | 0.74 (0.66,0.83) | 0.80 (0.71,0.90) | 0.87 (0.77,0.99) |
|  | Cheese | 0.56 (0.49,0.64) | 0.64 (0.56,0.73) | 0.75 (0.65,0.86) | 0.82 (0.71,0.94) |
|  | Yogurt | 0.33 (0.24,0.46) | 0.60 (0.45,0.81) | 0.82 (0.62,1.09) | 0.93 (0.70,1.24) |
| Poultry | Milk | 3.16 (2.09,4.79) | 1.07 (0.71,1.62) | 0.92 (0.61,1.39) | 1.13 (0.75,1.71) |
|  | Cheese | 2.83 (1.86,4.30) | 0.96 (0.63,1.45) | 0.86 (0.57,1.31) | 1.07 (0.70,1.62) |
|  | Yogurt | 1.37 (0.81,2.32) | 0.79 (0.48,1.31) | 0.88 (0.54,1.43) | 1.17 (0.72,1.90) |
| Stroke (2,544 cases/419,904.3 person-years) | | | | | |
| Total meat | Total dairy | 1.03 (0.93,1.13) | 0.88 (0.80,0.97) | 0.91 (0.82,1.01) | 0.92 (0.83,1.02) |
| Red meat | Milk | 1.08 (0.92,1.26) | 0.89 (0.77,1.04) | 0.91 (0.78,1.06) | 0.90 (0.77,1.05) |
|  | Cheese | 0.85 (0.71,1.01) | 0.78 (0.66,0.93) | 0.83 (0.70,0.99) | 0.83 (0.70,0.99) |
|  | Yogurt | 0.74 (0.51,1.07) | 0.92 (0.66,1.28) | 1.06 (0.76,1.48) | 1.09 (0.78,1.51) |
| Processed meat | Milk | 0.96 (0.82,1.12) | 0.80 (0.68,0.94) | 0.84 (0.72,0.99) | 0.87 (0.73,1.02) |
|  | Cheese | 0.76 (0.64,0.90) | 0.70 (0.59,0.84) | 0.77 (0.64,0.93) | 0.80 (0.66,0.96) |
|  | Yogurt | 0.69 (0.48,0.99) | 0.85 (0.61,1.19) | 1.01 (0.72,1.40) | 1.06 (0.76,1.47) |
| Poultry | Milk | 5.14 (2.97,8.88) | 1.62 (0.94,2.80) | 1.55 (0.90,2.69) | 1.60 (0.92,2.76) |
|  | Cheese | 4.90 (2.82,8.51) | 1.53 (0.88,2.66) | 1.49 (0.86,2.59) | 1.54 (0.88,2.66) |
|  | Yogurt | 4.18 (2.17,8.03) | 1.72 (0.91,3.24) | 1.84 (0.98,3.46) | 1.97 (1.05,3.68) |
| Ischemic stroke (1,113 cases/423,746.3 person years) | | | | | |
| Total meat | Total dairy | 1.09 (0.94,1.26) | 0.97 (0.83,1.13) | 1.02 (0.87,1.19) | 1.04 (0.89,1.22) |
| Red meat | Milk | 1.25 (0.98,1.60) | 1.07 (0.84,1.36) | 1.11 (0.87,1.41) | 1.13 (0.88,1.44) |
|  | Cheese | 0.93 (0.71,1.22) | 0.88 (0.67,1.14) | 0.96 (0.73,1.26) | 0.97 (0.74,1.28) |
|  | Yogurt | 0.91 (0.53,1.57) | 1.15 (0.70,1.89) | 1.37 (0.83,2.24) | 1.45 (0.89,2.36) |
| Processed meat | Milk | 0.99 (0.79,1.25) | 0.88 (0.69,1.12) | 0.94 (0.74,1.20) | 0.99 (0.77,1.27) |
|  | Cheese | 0.75 (0.58,0.97) | 0.73 (0.56,0.96) | 0.82 (0.62,1.09) | 0.86 (0.65,1.14) |
|  | Yogurt | 0.77 (0.46,1.31) | 0.99 (0.61,1.61) | 1.19 (0.73,1.94) | 1.30 (0.80,2.10) |
| Poultry | Milk | 4.00 (1.76,9.08) | 1.32 (0.58,2.99) | 1.35 (0.59,3.07) | 1.44 (0.64,3.25) |
|  | Cheese | 3.83 (1.68,8.77) | 1.26 (0.55,2.87) | 1.29 (0.56,2.96) | 1.37 (0.60,3.13) |
|  | Yogurt | 3.72 (1.41,9.83) | 1.59 (0.62,4.09) | 1.80 (0.70,4.62) | 2.03 (0.80,5.15) |
| Hemorrhagic stroke (449 cases/426,640.6 person-years) | | | | | |
| Total meat | Total dairy | 1.08 (0.85,1.37) | 0.96 (0.75,1.22) | 0.99 (0.77,1.27) | 0.97 (0.75,1.24) |
| Red meat | Milk | 1.15 (0.79,1.68) | 0.99 (0.68,1.44) | 0.99 (0.68,1.44) | 0.96 (0.66,1.39) |
|  | Cheese | 0.85 (0.56,1.29) | 0.80 (0.52,1.21) | 0.85 (0.55,1.29) | 0.82 (0.54,1.26) |
|  | Yogurt | 0.45 (0.16,1.21) | 0.57 (0.23,1.45) | 0.67 (0.27,1.68) | 0.64 (0.26,1.61) |
| Processed meat | Milk | 1.12 (0.77,1.64) | 0.98 (0.66,1.46) | 1.03 (0.69,1.55) | 1.01 (0.67,1.50) |
|  | Cheese | 0.83 (0.54,1.27) | 0.79 (0.51,1.23) | 0.89 (0.56,1.40) | 0.87 (0.55,1.37) |
|  | Yogurt | 0.44 (0.16,1.20) | 0.58 (0.23,1.45) | 0.70 (0.28,1.77) | 0.68 (0.27,1.71) |
| Poultry | Milk | 2.51 (0.71,8.85) | 1.10 (0.31,3.87) | 1.03 (0.29,3.61) | 1.03 (0.29,3.63) |
|  | Cheese | 2.32 (0.65,8.32) | 1.02 (0.28,3.63) | 0.96 (0.27,3.44) | 0.97 (0.27,3.46) |
|  | Yogurt | 1.14 (0.23,5.68) | 0.67 (0.14,3.14) | 0.72 (0.16,3.33) | 0.72 (0.16,3.32) |

^1^All HRs and 95% CIs were derived from Cox proportional hazard regression models. Age, sex, energy-adjusted model included age, sex, and total energy intake. Sociodemographic, lifestyle and energy-adjusted model included additional adjustment for educational level, physical activity, smoking status, alcohol intake, fiber intake, fruit and vegetables and hormone replacement therapy (females only). Sociodemographic, lifestyle, energy and cardiometabolic characteristics-adjusted model was further adjusted for body mass index, waist circumference, baseline hypertension, baseline hypercholesterolemia, baseline diabetes mellitus. Abbreviations: CAD, coronary artery disease, CI, confidence interval; CVD, cardiovascular disease; En%, percentage of total energy intake; EPIC, European Prospective Investigation into Cancer, and Nutrition; HR, hazard ratio; SFA, saturated fatty acids

#

# **SUPPLEMENTARY TABLE 11** Hazard ratios and 95% confidence intervals for the risk of overall and subtypes of cardiovascular disease associated with saturated fatty acid intakes from total and types of meat by total and types of dairy in the EPIC-Norfolk Study (n=21,841)^1^

| Outcome and adjustment models | Hazard ratios (95% confidence intervals) for the effect estimates (per 2.5 en%) | | | | | | | |
| --- | --- | --- | --- | --- | --- | --- | --- | --- |
|  | Total meat | Red meat | Processed meat | Poultry | Total dairy | Milk | Cheese | Yogurt |
| CVD |  |  |  |  |  |  |  |  |
| Multivariable | 1.07 (1.01,1.14) | 1.06 (0.97,1.17) | 1.16 (1.05,1.27) | 0.80 (0.57,1.14) | 0.98 (0.95,1.01) | 1.00 (0.96,1.04) | 0.91 (0.85,0.98) | 1.04 (0.84,1.30) |
| + dietary factors | 1.11 (1.01,1.22) | 1.01 (0.89,1.15) | 1.18 (1.05,1.33) | 0.70 (0.45,1.08) | 1.01 (0.94,1.07) | 1.04 (0.98,1.10) | 0.91 (0.84,0.99) | 1.09 (0.88,1.37) |
| CAD |  |  |  |  |  |  |  |  |
| Multivariable | 1.05 (0.98,1.12) | 1.03 (0.92,1.15) | 1.12 (1.00,1.25) | 0.88 (0.58,1.32) | 0.96 (0.92,1.00) | 0.97 (0.92,1.02) | 0.91 (0.83,0.99) | 1.03 (0.78,1.34) |
| + dietary factors | 1.13 (1.01,1.26) | 1.03 (0.89,1.19) | 1.19 (1.04,1.37) | 0.80 (0.48,1.35) | 1.00 (0.92,1.08) | 1.03 (0.96,1.10) | 0.93 (0.84,1.02) | 1.09 (0.83,1.43) |
| Stroke |  |  |  |  |  |  |  |  |
| Multivariable | 1.11 (1.01,1.22) | 1.14 (0.99,1.31) | 1.19 (1.02,1.38) | 0.63 (0.36,1.08) | 1.02 (0.97,1.07) | 1.03 (0.97,1.09) | 0.94 (0.84,1.05) | 1.23 (0.91,1.67) |
| + dietary factors | 1.06 (0.92,1.22) | 1.00 (0.82,1.20) | 1.11 (0.92,1.34) | 0.49 (0.25,0.96) | 1.00 (0.91,1.11) | 1.01 (0.93,1.10) | 0.91 (0.81,1.03) | 1.27 (0.93,1.73) |
| Ischemic stroke |  |  |  |  |  |  |  |  |
| Multivariable | 1.01 (0.87,1.16) | 0.97 (0.77,1.21) | 1.09 (0.86,1.38) | 0.70 (0.31,1.58) | 1.05 (0.98,1.14) | 1.08 (0.99,1.19) | 0.94 (0.79,1.10) | 1.41 (0.91,2.20) |
| + dietary factors | 1.05 (0.85,1.31) | 0.93 (0.69,1.25) | 1.18 (0.89,1.58) | 0.75 (0.27,2.08) | 1.12 (0.96,1.31) | 1.11 (0.98,1.27) | 0.89 (0.74,1.06) | 1.44 (0.92,2.25) |
| Hemorrhagic stroke | |  |  |  |  |  |  |  |
| Multivariable | 1.06 (0.85,1.32) | 1.11 (0.79,1.55) | 1.05 (0.72,1.53) | 0.97 (0.28,3.41) | 1.02 (0.91,1.16) | 1.05 (0.91,1.22) | 0.90 (0.69,1.18) | 0.70 (0.29,1.66) |
| + dietary factors | 1.19 (0.83,1.70) | 1.16 (0.72,1.85) | 1.16 (0.73,1.85) | 0.74 (0.15,3.61) | 1.31 (0.98,1.74) | 1.19 (0.96,1.49) | 0.91 (0.68,1.23) | 0.66 (0.27,1.62) |

^1^All HRs and 95% CIs were derived from Cox proportional hazard regression models. Multivariable adjusted model included adjustments for age, sex, total energy intake, educational level, physical activity, smoking status, alcohol intake, fiber intake, fruit and vegetables, hormone replacement therapy (females only), body mass index, waist circumference, baseline hypertension, baseline hypercholesterolemia, baseline diabetes mellitus. Most adjusted model included adjustments for age, sex, educational level, physical activity, smoking status, alcohol intake, fiber intake, fruit and vegetables, hormone replacement therapy (females only), dietary cholesterol, trans fatty acids, protein, carbohydrate, polyunsaturated fatty acids, monounsaturated fatty acids, saturated fatty acids from other foods, body mass index, waist circumference, baseline hypertension, baseline hypercholesterolemia, baseline diabetes mellitus. Saturated fatty acids from all meat and dairy sources were mutually adjusted for each other. N of CVD cases: 5902 over person-years of 395,854.5. N of CAD cases: 4215 over person years of 401,846.7. N of stroke cases: 2544 over person years of 419,904.3. N of ischemic stroke cases: 1113 over person years of 423,746.3. N of hemorrhagic stroke cases: 449 over person-years of 426,640.6. Abbreviations: CAD, coronary artery disease; CI, confidence interval; CVD, cardiovascular disease; En%, percentage of total energy intake; EPIC, European Prospective Investigation into Cancer, and Nutrition; HR, hazard ratio; SFA, saturated fatty acids

# **SUPPLEMENTARY TABLE 12** Hazard ratios and 95% confidence intervals for risk of cardiovascular disease associated with the modeled replacement of saturated fatty acids from total and types of meat by total and types of dairy in the EPIC-Norfolk Study (n=21,841): Sensitivity analyses^1^

| Sensitivity analyses | SFA from food to be replaced | SFA from replacement food | Hazard ratios and 95% confidence intervals  for replacement effects (per 2.5 en%) estimated from the most adjusted models | | | | |
| --- | --- | --- | --- | --- | --- | --- | --- |
|  |  |  | CVD | CAD | Stroke | Ischemic stroke | Hemorrhagic stroke |
| Excluding incident cases in the first two years  of follow-up | N cases / N total | | 5763/21,702 | 4106/21,732 | 2513/21,810 | 1102/21,830 | 443/21,835 |
|  | Total meat | Total dairy | 0.89 (0.82,0.96) | 0.87 (0.79,0.96) | 0.93 (0.82,1.05) | 1.02 (0.85,1.23) | 0.96 (0.71,1.29) |
|  | Red meat | Milk | 0.93 (0.82,1.04) | 0.91 (0.79,1.05) | 0.91 (0.77,1.09) | 1.14 (0.86,1.50) | 0.95 (0.62,1.45) |
|  |  | Cheese | 0.85 (0.75,0.97) | 0.85 (0.73,0.99) | 0.86 (0.71,1.04) | 0.99 (0.73,1.33) | 0.83 (0.52,1.31) |
|  |  | Yogurt | 0.97 (0.76,1.24) | 0.95 (0.70,1.28) | 1.15 (0.82,1.61) | 1.50 (0.91,2.48) | 0.59 (0.23,1.53) |
|  | Processed meat | Milk | 0.85 (0.75,0.95) | 0.82 (0.72,0.94) | 0.88 (0.74,1.06) | 0.95 (0.73,1.25) | 0.99 (0.64,1.54) |
|  |  | Cheese | 0.78 (0.68,0.88) | 0.76 (0.66,0.89) | 0.83 (0.68,1.01) | 0.83 (0.62,1.13) | 0.87 (0.53,1.41) |
|  |  | Yogurt | 0.90 (0.71,1.15) | 0.87 (0.65,1.17) | 1.11 (0.79,1.56) | 1.29 (0.78,2.12) | 0.62 (0.24,1.62) |
|  | Poultry | Milk | 1.49 (0.99,2.26) | 1.31 (0.80,2.14) | 2.02 (1.07,3.83) | 1.48 (0.57,3.86) | 1.81 (0.41,8.04) |
|  |  | Cheese | 1.39 (0.92,2.10) | 1.24 (0.76,2.02) | 1.94 (1.02,3.67) | 1.29 (0.50,3.38) | 1.53 (0.34,6.81) |
|  |  | Yogurt | 1.55 (0.98,2.47) | 1.35 (0.78,2.35) | 2.54 (1.26,5.13) | 1.95 (0.68,5.58) | 1.06 (0.19,5.88) |
|  |  |  |  |  |  |  |  |
| Excluding participants with self-reported diabetes mellitus or cancer at baseline | N cases / N total |  | 5354/20,268 | 3832/20,268 | 2290/20,268 | 994/20,268 | 409/20,268 |
|  | Total meat | Total dairy | 0.91 (0.84,0.99) | 0.90 (0.81,0.99) | 0.93 (0.81,1.05) | 1.02 (0.84,1.24) | 0.91 (0.67,1.24) |
|  | Red meat | Milk | 0.95 (0.84,1.07) | 0.94 (0.81,1.08) | 0.91 (0.76,1.10) | 1.11 (0.83,1.48) | 0.90 (0.58,1.39) |
|  |  | Cheese | 0.87 (0.76,1.00) | 0.87 (0.74,1.01) | 0.85 (0.70,1.04) | 0.94 (0.69,1.29) | 0.72 (0.45,1.17) |
|  |  | Yogurt | 1.04 (0.80,1.33) | 1.06 (0.79,1.44) | 1.07 (0.74,1.54) | 1.49 (0.87,2.55) | 0.50 (0.18,1.40) |
|  | Processed meat | Milk | 0.87 (0.77,0.98) | 0.85 (0.74,0.98) | 0.95 (0.73,1.25) | 0.99 (0.74,1.32) | 1.00 (0.63,1.57) |
|  |  | Cheese | 0.80 (0.70,0.91) | 0.78 (0.67,0.92) | 0.83 (0.62,1.13) | 0.84 (0.61,1.16) | 0.80 (0.48,1.34) |
|  |  | Yogurt | 0.96 (0.75,1.24) | 0.98 (0.73,1.32) | 1.29 (0.78,2.12) | 1.35 (0.79,2.31) | 0.56 (0.20,1.57) |
|  | Poultry | Milk | 1.47 (0.95,2.28) | 1.39 (0.83,2.34) | 1.80 (0.91,3.57) | 1.35 (0.48,3.81) | 1.51 (0.32,7.16) |
|  |  | Cheese | 1.36 (0.88,2.12) | 1.30 (0.77,2.20) | 1.72 (0.87,3.40) | 1.15 (0.41,3.26) | 1.17 (0.24,5.55) |
|  |  | Yogurt | 1.59 (0.97,2.60) | 1.57 (0.87,2.81) | 2.11 (0.99,4.48) | 1.79 (0.57,5.59) | 0.77 (0.13,4.70) |
|  |  |  |  |  |  |  |  |
| Age as underlying time scale | N cases / N total |  | 5,902/21,841 | 4215/21,841 | 2544/21,841 | 1113/21,841 | 449/21,841 |
|  | Total meat | Total dairy | 0.89 (0.82,0.96) | 0.88 (0.80,0.96) | 0.92 (0.81,1.04) | 1.02 (0.85,1.23) | 0.95 (0.70,1.27) |
|  | Red meat | Milk | 0.93 (0.83,1.04) | 0.91 (0.79,1.04) | 0.91 (0.76,1.09) | 1.09 (0.83,1.42) | 0.95 (0.63,1.46) |
|  |  | Cheese | 0.86 (0.76,0.97) | 0.86 (0.74,0.99) | 0.86 (0.71,1.04) | 1.13 (0.86,1.48) | 0.82 (0.52,1.30) |
|  |  | Yogurt | 0.98 (0.77,1.25) | 0.96 (0.72,1.29) | 1.13 (0.81,1.59) | 0.99 (0.73,1.33) | 0.59 (0.23,1.52) |
|  | Processed meat | Milk | 0.84 (0.75,0.94) | 0.83 (0.73,0.95) | 0.88 (0.73,1.05) | 0.96 (0.74,1.26) | 0.96 (0.62,1.47) |
|  |  | Cheese | 0.77 (0.68,0.88) | 0.78 (0.67,0.90) | 0.82 (0.67,1.00) | 0.85 (0.63,1.15) | 0.83 (0.51,1.34) |
|  |  | Yogurt | 0.91 (0.71,1.15) | 0.89 (0.66,1.19) | 1.09 (0.78,1.53) | 1.32 (0.80,2.16) | 0.60 (0.23,1.55) |
|  | Poultry | Milk | 1.54 (1.02,2.31) | 1.36 (0.84,2.20) | 2.03 (1.08,3.82) | 1.43 (0.55,3.70) | 1.88 (0.43,8.28) |
|  |  | Cheese | 1.43 (0.95,2.15) | 1.29 (0.79,2.09) | 1.94 (1.03,3.65) | 1.26 (0.49,3.26) | 1.57 (0.35,6.90) |
|  |  | Yogurt | 1.61 (1.01,2.54) | 1.42 (0.82,2.45) | 2.52 (1.25,5.06) | 1.91 (0.67,5.40) | 1.09 (0.20,6.00) |

^1^All HRs and 95% CIs were derived from Cox proportional hazard regression models. Most adjusted model included age, sex, educational level, physical activity, smoking status, alcohol intake, fiber intake, fruit and vegetables, hormone replacement therapy (females only), dietary cholesterol, trans fatty acids, protein, carbohydrate, polyunsaturated fatty acids, monounsaturated fatty acids, saturated fatty acids from other foods, body mass index, waist circumference, baseline hypertension, baseline hypercholesterolemia, baseline diabetes mellitus. Abbreviations: CAD, coronary artery disease; CI, confidence interval; CVD, cardiovascular disease; En%, percentage of total energy intake; EPIC, European Prospective Investigation into Cancer, and Nutrition; HR, hazard ratio; SFA, saturated fatty acids

# **SUPPLEMENTARY TABLE 13** Hazard ratios and 95% confidence intervals for risk of overall and subtypes of cardiovascular disease associated with the Modeled replacement of saturated fatty acids from total meat by total dairy, stratified by potential effect modifiers, in the EPIC-Norfolk Study (n=21,841)^1^

| Replacement of SFA from total meat by SFA from total dairy  (per 2.5en%) | Outcome | Strata | Cases/total | Most adjusted model | P interaction |
| --- | --- | --- | --- | --- | --- |
|  |  |  |  | HR (95% CI) |  |
| Interaction by sex | CVD | Male | 3105/9524 | 0.87 (0.78,0.97) | 0.4716 |
|  |  | Female | 2797/12317 | 0.95 (0.84,1.07) |  |
|  | CAD | Male | 2408/9524 | 0.90 (0.80,1.01) | 0.9388 |
|  |  | Female | 1807/12317 | 0.87 (0.74,1.02) |  |
|  | Stroke | Male | 1146/9524 | 0.81 (0.68,0.96) | 0.3581 |
|  |  | Female | 1398/12317 | 1.07 (0.89,1.28) |  |
|  | Ischemic stroke | Male | 529/9524 | 0.88 (0.69,1.14) | 0.1494 |
|  |  | Female | 584/12317 | 1.30 (0.98,1.73) |  |
|  | Hemorrhagic stroke | Male | 200/9524 | 0.80 (0.52,1.22) | 0.1626 |
|  |  | Female | 249/12317 | 1.09 (0.72,1.65) |  |
| Interaction by age | CVD | <60 years | 1937/12261 | 0.92 (0.80,1.05) | 0.3674 |
|  |  | ≥60 years | 3965/9580 | 0.89 (0.80, 0.98) |  |
|  | CAD | <60 years | 1500/12261 | 0.95 (0.82,1.11) | 0.6279 |
|  |  | ≥60 years | 2715/9580 | 0.85 (0.75,0.95) |  |
|  | Stroke | <60 years | 609/12261 | 0.95 (0.82,1.11) | 0.3348 |
|  |  | ≥60 years | 1935/9580 | 0.97 (0.84,1.13) |  |
|  | Ischemic stroke | <60 years | 295/12261 | 1.15 (0.83,1.59) | 0.7508 |
|  |  | ≥60 years | 818/9580 | 0.98 (0.79,1.24) |  |
|  | Hemorrhagic stroke | <60 years | 132/12261 | 0.61 (0.38,0.99) | 0.2654 |
|  |  | ≥60 years | 317/9580 | 1.22 (0.84,1.77) |  |
| Interaction by baseline hypertension/hypercholesterolemia/  diabetes mellitus | CVD | No/unknown | 1995/11075 | 0.93 (0.82,1.06) | 0.1479 |
|  |  | Yes | 3907/10766 | 0.88 (0.80, 0.97) |  |
|  | CAD | No/unknown | 1413/11075 | 0.87 (0.74,1.02) | 0.0579 |
|  |  | Yes | 2802/10766 | 0.90 (0.80,1.01) |  |
|  | Stroke | No/unknown | 803/11075 | 1.08 (0.87,1.34) | 0.0492 |
|  |  | Yes | 1741/10766 | 0.86 (0.74,1.00) |  |
|  | Ischemic stroke | No/unknown | 341/11075 | 1.17 (0.84,1.63) | 0.2841 |
|  |  | Yes | 772/10766 | 0.97 (0.78,1.22) |  |
|  | Hemorrhagic stroke | No/unknown | 159/11075 | 0.94 (0.58,1.51) | 0.9828 |
|  |  | Yes | 290/10766 | 0.98 (0.67,1.42) |  |

^1^All HRs and 95% CIs were derived from Cox proportional hazard regression models. Most adjusted model included age, sex, educational level, physical activity, smoking status, alcohol intake, fiber intake, fruit and vegetables, hormone replacement therapy (females only), dietary cholesterol, trans fatty acids, protein, carbohydrate, polyunsaturated fatty acids, monounsaturated fatty acids, saturated fatty acids from other foods, body mass index, waist circumference, baseline hypertension, baseline hypercholesterolemia, baseline diabetes mellitus. Abbreviations: CAD, coronary artery disease; CI, confidence interval; CVD, cardiovascular disease; En%, percentage of total energy intake; EPIC, European Prospective Investigation into Cancer, and Nutrition; HR, hazard ratio; SFA, saturated fatty acids

25,630 participants eligible for longitudinal analysis

Excluded:
Participants with a prevalent CVD or no data on prevalent CVD (n=2563)

23,067 participants

Excluded:
Participants with missing dietary data (n=793)

22,274 participants

Excluded:
Participants with implausible energy intakes^1^ (n=222)
Participants with missing covariate data (n=211):
Smoking (n=168), physical activity (n=1), waist circumference (n=16), BMI (n=18), baseline diabetes mellitus (n=8)

21,841 participants for analyses of incident CVD

**SUPPLEMENTARY FIGURE 1** Flowchart of participant selection for the analyses on the estimated effect of isoenergetic replacement of SFA from meat by SFA from dairy on the incidence of CVD in adults of the EPIC-Norfolk Study^1^

^1^Potential under- or over-reporters of energy intake were excluded, based on the bottom or top (0.5%) percentiles (Energy intake /basal metabolic rate<0.61 or >2.81). Abbreviations: CVD, cardiovascular disease; EPIC, European Prospective Investigation into Cancer, and Nutrition; SFA, saturated fatty acids


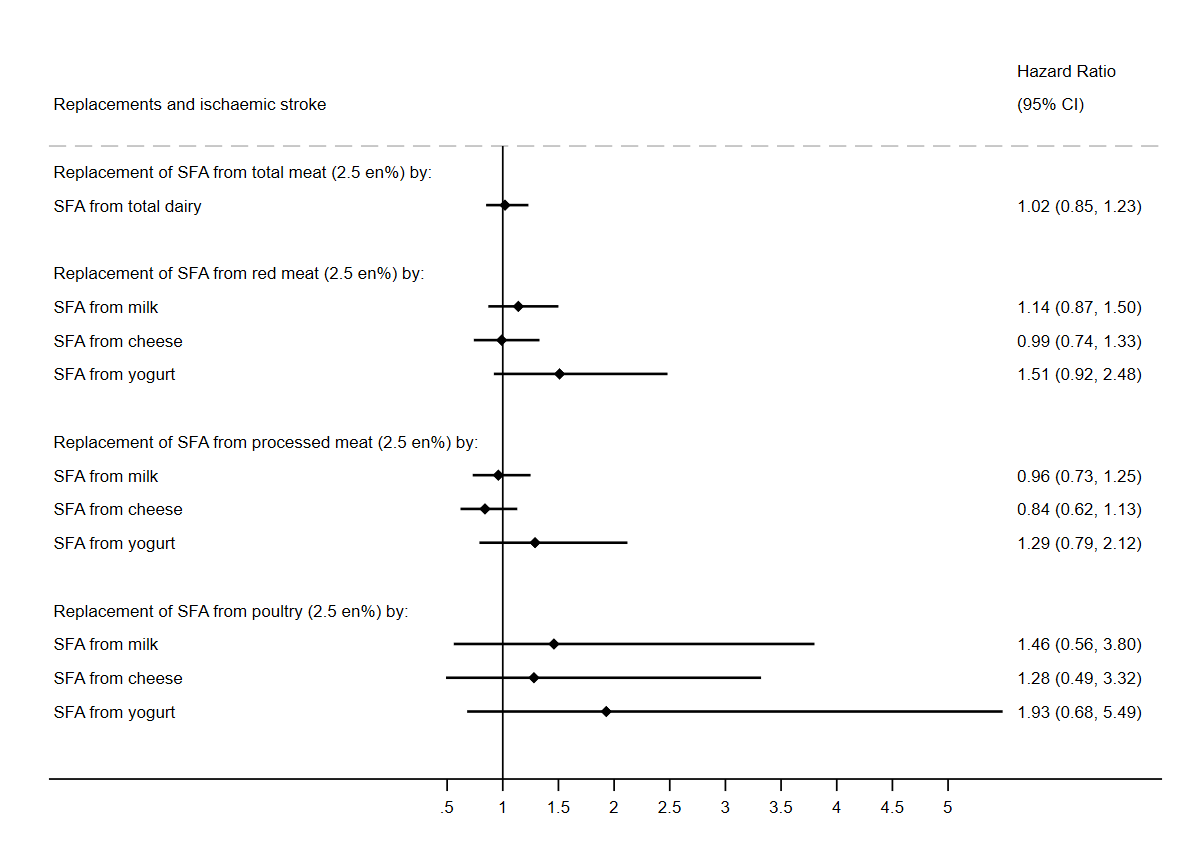


# **SUPPLEMENTARY FIGURE 2** Modeled replacement of SFA from total, red, processed or poultry meat (2.5% of total energy) by the equivalent from total and types of dairy in relation to incident ischemic stroke: the EPIC-Norfolk Study(n=21,841)^1^

^1^Multivariable adjusted Cox model included age, sex, educational level, physical activity, smoking status, alcohol intake, fiber intake, fruit and vegetables, hormone replacement therapy (females only), dietary cholesterol, trans fatty acids, protein, carbohydrate, polyunsaturated fatty acids, monounsaturated fatty acids, saturated fatty acids from other foods, body mass index, waist circumference, baseline hypertension, baseline hypercholesterolemia, baseline diabetes mellitus. N of ischemic stroke cases: 1113 over person years of 423,746.3. Abbreviations: En%, percentage of total energy intake; EPIC, European Prospective Investigation into Cancer and Nutrition; SFA, saturated fatty acids.


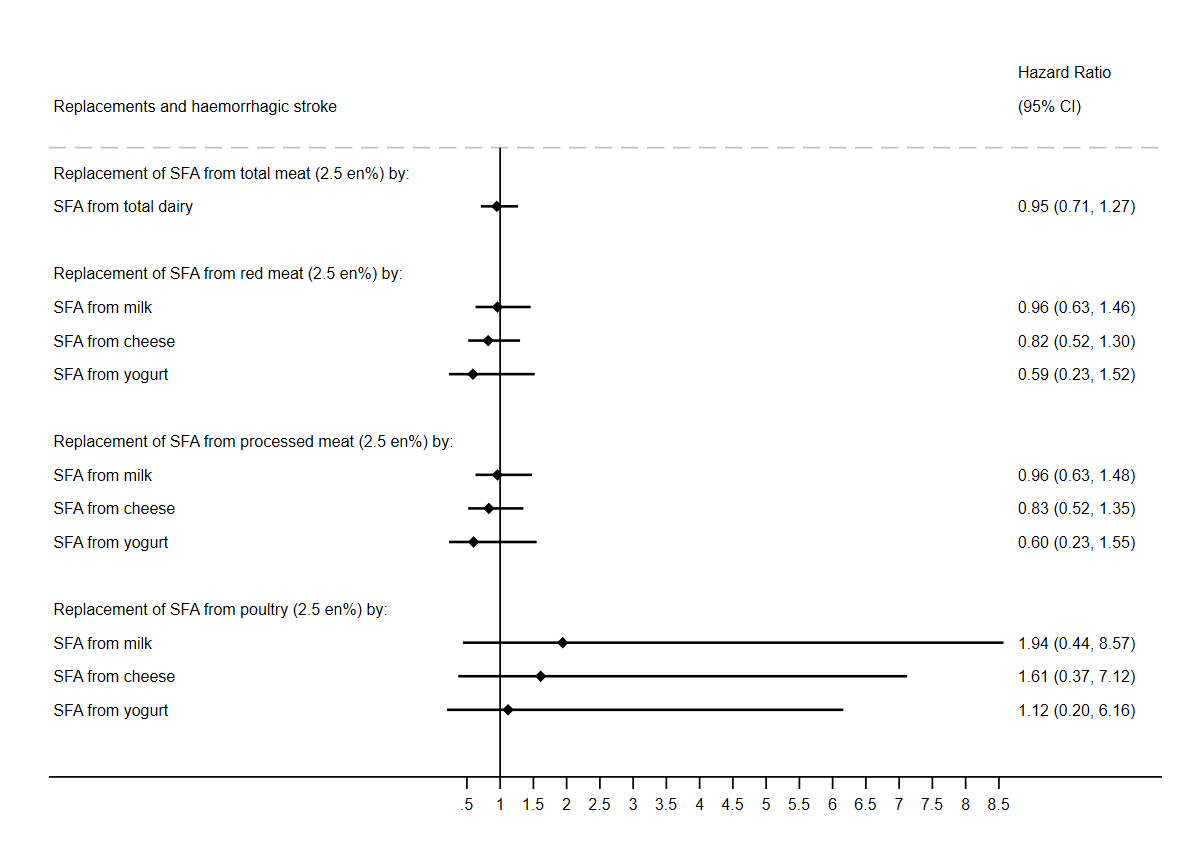


# **SUPPLEMENTARY FIGURE 3** Modeled replacement of SFA from total, red, processed or poultry meat (2.5% of total energy) by the equivalent from total and types of dairy in relation to incident hemorrhagic stroke: the EPIC-Norfolk Study(n=21,841)^1^

^1^Multivariable adjusted Cox model included age, sex, educational level, physical activity, smoking status, alcohol intake, fiber intake, fruit and vegetables, hormone replacement therapy (females only), dietary cholesterol, trans fatty acids, protein, carbohydrate, polyunsaturated fatty acids, monounsaturated fatty acids, saturated fatty acids from other foods, body mass index, waist circumference, baseline hypertension, baseline hypercholesterolemia, baseline diabetes mellitus. N of hemorrhagic stroke cases: 449 over person-years of 426,640.6. Abbreviations: En%, percentage of total energy intake; EPIC, European Prospective Investigation into Cancer and Nutrition; SFA, saturated fatty acids.
